# Detecting the symplesiomorphy trap: a multigene phylogenetic analysis of terebelliform annelids

**DOI:** 10.1186/1471-2148-11-369

**Published:** 2011-12-20

**Authors:** Min Zhong, Benjamin Hansen, Maximilian Nesnidal, Anja Golombek, Kenneth M Halanych, Torsten H Struck

**Affiliations:** 1 Auburn University, Department of Biological Sciences, 101 Life Science Building, Auburn, AL 36849, USA; 2 University of Osnabrück, FB05 Biology/Chemistry, AG Zoology, Barbarastr. 11, 49069 Osnabrück, Germany

## Abstract

**Background:**

For phylogenetic reconstructions, conflict in signal is a potential problem for tree reconstruction. For instance, molecular data from different cellular components, such as the mitochondrion and nucleus, may be inconsistent with each other. Mammalian studies provide one such case of conflict where mitochondrial data, which display compositional biases, support the Marsupionta hypothesis, but nuclear data confirm the Theria hypothesis. Most observations of compositional biases in tree reconstruction have focused on lineages with different composition than the majority of the lineages under analysis. However in some situations, the position of taxa that lack compositional bias may be influenced rather than the position of taxa that possess compositional bias. This situation is due to apparent symplesiomorphic characters and known as "the symplesiomorphy trap".

**Results:**

Herein, we report an example of the sympleisomorphy trap and how to detect it. Worms within Terebelliformia (sensu Rouse & Pleijel 2001) are mainly tube-dwelling annelids comprising five 'families': Alvinellidae, Ampharetidae, Terebellidae, Trichobranchidae and Pectinariidae. Using mitochondrial genomic data, as well as data from the nuclear 18S, 28S rDNA and elongation factor-1α genes, we revealed incongruence between mitochondrial and nuclear data regarding the placement of Trichobranchidae. Mitochondrial data favored a sister relationship between Terebellidae and Trichobranchidae, but nuclear data placed Trichobranchidae as sister to an Ampharetidae/Alvinellidae clade. Both positions have been proposed based on morphological data.

**Conclusions:**

Our investigation revealed that mitochondrial data of Ampharetidae and Alvinellidae exhibited strong compositional biases. However, these biases resulted in a misplacement of Trichobranchidae, rather than Alvinellidae and Ampharetidae. Herein, we document that Trichobranchidae was apparently caught in the symplesiomorphy trap suggesting that in certain situations even homologies can be misleading.

## Background

**Figure 1:**
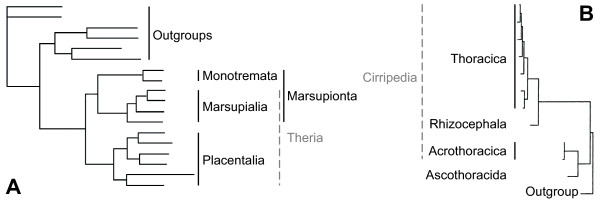
**Examples of misplacements**. (A) Marsupialia within Mammalia based on mitochondrial data [modified from [15]] and (B) Ascothoracida within Cirripedia [modified from [74]]. Only more inclusive taxonomic units are indicated for reasons of simplicity.

The amount of data used in phylogenetic reconstructions has been steadily increasing during the past decade [e.g., [1-4]], and phylogenies based on multiple datasets (i.e., partitions) are now common. However, analyses based on different partitions do not always result in congruent phylogenetic reconstructions. Molecular evolutionary events such as gene duplication, horizontal gene transfer, heterotachy, gene extinction, long-branch attraction, saturation and model misspecifications can cause inferred gene trees to differ from species trees. For example, incongruence regarding phylogenetic placement of taxa can occur between mitochondrial and nuclear data [e.g., [5]]. In the case of mammals, mitochondrial data strongly support the Marsupionta hypothesis placing Marsupialia as sister to Monotremata (Figure 1A) [6-11], whereas the Theria hypothesis, which places Marsupialia with Placentalia, has been strongly supported by both morphological and nuclear data [e.g., [12-14]]. Phillips and Penny [15] showed that strong compositional biases in pyrimidine and purine frequencies in mitochondrial genomes of Marsupialia and Monotremata provided support for the Marsupionta hypothesis. However, both partitioning the dataset and to a lesser degree RY coding were able to effectively minimize artificial signal. In general, taxa affected by biases such as increased substitutions rates, heterotachy, etc., are the ones misplaced in phylogenetic analyses. However, biases may also influence the placement of unbiased taxa. In the case of the symplesiomorphy trap [16], a paraphyletic assemblage of taxa is grouped together as monophyletic based on the possession of symplesiomorphic characters, which are mistakenly assumed to be apomorphic. The symplesiomorphy trap has been characterized as a special class of long-branch attraction by Wägele & Mayer [17].

This problem is common for morphological data and several instances are known. One well-known annelid example is the position of Clitellata as sister to Polychaeta due to the lack of typical polychaete characters such as parapodia and nuchal organs [18]. However, molecular data clearly place Clitellata within polychaetes [e.g., [2,3,19]]. In theory, the symplesiomorphy trap is not restricted to morphological data, but can also apply to sequence data [16]. However, studies addressing this problem in molecular data are scarce because detection of the trap is not straightforward. First, the misplaced taxa are not themselves affected by compositional biases or increased substitution rates. Second, support for monophyly of misplaced taxa is based on apomorphies for a higher taxonomic unit and hence not artificial. Third, knowledge of the 'true' phylogeny is needed to directly detect the symplesiomorphy trap. Typically, detection of the trap occurs indirectly by excluding other possibilities of incongruence and revealing characteristic signatures in the data. For example, Wägele and Mayer's [17] study showed that misplacement of Acrothoracica barnacles in a 18S parsimony analysis was due to symplesiomorphic characters shared exclusively by Ascothoracida (a non-barnacle outgroup) and Acrothoracica (Figure 1B). These characters overwhelmed the phylogenetic signal for the monophyly of Cirripedia. This phenomenon is known as the symplesiomorphy trap.

Here we report another instance of the symplesiomorphy trap in molecular data discovered while examining Terebelliformia (Annelida) phylogeny. Terebelliform worms [sensu [20]] are typically tube-dwelling annelids, found in diverse marine habitats, including intertidal, deep-sea and even hydrothermal vent areas. Terebelliformia include about 800 species within five 'families': Alvinellidae, Ampharetidae, Terebellidae, Trichobranchidae and Pectinariidae [20-22]. Based on thorough investigations using data partitioning, topology tests, removal and addition of taxa, spectral analyses, detection of compositional biases, models of non-stationary sequence evolution, and recoding of characters, we were able to pinpoint the source of the incongruence between mitochondrial and nuclear data and relate it to the symplesiomorphy trap. Ampharetidae and Alvinellidae exhibit strong compositional biases in their mitochondrial genomes. However, these biases affect placement of Trichobranchidae and Terebellidae rather than Ampharetidae and Alvinellidae.

## Methods

### Sample and Data Collection

Table 1 lists taxa, gene sequences, GenBank accession numbers and sample locations used in this study. Upon collection, tissue samples were preserved in >70% non-denatured ethanol or frozen at -80°C. Genomic DNA was extracted using the DNeasy Tissue Kit (Qiagen, Hilden, Germany) according to the manufacturer's instructions. Mitochondrial genomes were amplified following Zhong et al. [23] in four overlapping segments using species-specific primers (for more details see Additional File 1). Amplification and sequencing of nuclear 18S and 28S genes was carried out using protocols described by Struck et al. [24]. Presence of PCR products were confirmed on a 1% agarose gel and purified with the QIAquick PCR Purification or QIAquick Gel Extraction kit (Qiagen, Hilden, Germany). When necessary, PCR products were size-selected on agarose gels and/or cloned using pGEM^®^-T Easy Vector System (Promega, Madison, WI, USA) or StrataClone™ PCR Cloning Kit (Stratagene, La Jolla, CA, USA). A CEQ™ 8000 Genetic Analysis System (Beckman Coulter, Fullerton, CA, USA) or ABI Prism 377 Automatic Sequencer (Perkin Elmer, Shelton, CT, USA) was used for bidirectional sequencing of all PCR products.

**Table 1:** Taxa used in phylogenetic analyses with 17 taxa.

Taxon	Species	mtDNA^1^	18S rDNA	28S rDNA	**EF1**α**^2^**	Locality^3^
**Terebelliformia**						

Ampharetidae	*Eclysippe vanelli *	EU239687	JN936467	JN936489		63°30.84'N/10°25.01'E Storgrunnen (Norway)
	*Auchenoplax crinita*	FJ976041	DQ790077	DQ790026	DQ813352	39°53.88'N/69°39.64'W Southern New England (USA)
Pectinariidae	*Pectinaria gouldi *	FJ976040	DQ790091	DQ790054		41°37.91'N/70°53.34'W Egypt Lane, Fairhaven, MA (USA)
	*Pectinaria koreni*				DQ813388	
Terebellidae	*Pista cristata*	EU239688	AY611461	DQ790057	DQ813391	
Trichobranchidae	*Terebellides stroemi*	EU236701	DQ790094	DQ790066		
	*Terebellides sp*.				DQ813404	
Alvinellidae	*Paralvinella sulfincola*	FJ976042	JN936461			47°57.001'N/129°05.851'W Juan de Fuca (Canada)
	*Paralvinella palmiformis*			JN936479		47°56.947'N/129°05.878'W Juan de Fuca (Canada)
	*Paralvinella hessleri*				DQ813385	

**Outgroups**						

Siboglinidae	*Galathealinum brachiosum*	AF178679	AF168738			
	*Siboglinum fiordicum*			DQ790061	DQ813398	
	*Riftia pachyptila*	AY741662	AF168739	Z21534	DQ813394	
Clitellata	*Helobdella robusta*	AF178680				
	*Helobdella triserialis*		AY962435			
	*Hirudo medicinalis*			AY364866	U90063	
	*Lumbricus terrestris*	NC_001673	AJ272183			
	*Lumbricus sp*.			DQ790041	DQ813373	
Nereididae	*Platynereis dumerilii*	NC_000931	EF117897			
	*Nereis succinea*			AY210464		
	*Nereis virens*				U90064	
Echiura	*Urechis caupo*	AY619711	AF342805		DQ813410	
	*Arhynchite pugettensis*			AY210455		
Nephtyidae	*Nephtys longosetosa*		DQ790082	DQ790042		
	*Nephtys *sp.	EU293739			DQ813376	
Sipuncula	*Phascolopsis gouldi*	AF374337	AF123306	AF342795	AF063421	
Orbiniidae	*Orbinia latreillii*	AY961084	AY532355			
	*Orbinia swani*		DQ790087	DQ790048		
	*Orbinia michaelseni*				DQ813381	
	*Scoloplos *cf. *armiger*	DQ517436	AY340443	AY366515		
Maldanidae	*Clymenella torquata*	AY741661		DQ790030	DQ813356	
	*Clymenura clypeata*		AF448152			

Accession numbers of determined sequences are in bold.

^1^mtDNA = mitochondrial genome

^2^EF1α = elongation factor 1α

^3^for locality information on available data see original source

### Genomic Assembly and Gene Identification

Sequences were edited and aligned using DNASTAR™ Lasergene programs SeqMan and MegAlign [25]. Protein-coding genes and ribosomal RNA genes were identified by BLAST [26]. All tRNA genes were identified using tRNAscan-SE web server [http://lowelab.ucsc.edu/tRNAscan-SE/, [27]] under default settings and source = "mito/chloroplast", or by hand based on their potential secondary structures and anticodon sequences.

### Datasets

Datasets consisted of mitochondrial and nuclear data. All alignments are available at TreeBASE http://www.treebase.org. Seventeen available annelid mitochondrial genomes with about 50% coverage or greater were used for the phylogenetic analyses (Table 1). The alignment of Zhong et al. [23] was employed with the addition of *Nephtys *sp.*, Pectinaria gouldi, Paralvinella sulfincola *and *Auchenoplax crinita*. Because we were interested in relationships within Terebelliformia, we deleted the mitochondrial data of *Katharina *(Mollusca) and *Terebratalia *(Brachiopoda) and used all other annelids as outgroup taxa.

Both nucleotide and amino acid datasets were created for mitochondrial phylogenetic analyses. In the nucleotide dataset, all protein-coding genes (except for *atp6*, *atp8 *and *nad6 *genes which exhibit high variability) and the two rRNA genes (*mLSU *and *mSSU*) were included. Clustal X [28] under default settings was used to align rRNA genes. Gblocks 0.91b [29] was used to identify ambiguous aligned regions in the rRNA genes. These regions and the 3^rd ^positions of protein-coding genes, which are saturated with substitutions for family-level analyses, were excluded from the analyses with the aid of MacClade4.08 [30] and Se-Al v2.0a11 [31]. The amino acid dataset was created from the aligned nucleotide dataset by translation of protein-coding genes with the *Drosophila *mitochondrial genetic code and exclusion of rRNA genes. The mitochondrial nucleotide and amino acid datasets comprised 6,287 and 2,990 positions, respectively.

Additionally, a combined data matrix was constructed with the addition of 18S, 28S and EF-1α sequences to the mitochondrial data for the above 17 taxa (Table 1). Because we employed data from GenBank and collected data in two different laboratories (Univ. of Osnabrück and Auburn Univ.), in some cases we concatenated data from as closely related species as possible to generate Operational Taxonomic Units (OTUs) with a more complete coverage (see Table 1). Sequences were aligned as above. Due to the addition of nuclear data, the combined datasets comprised 11,813 nucleotide and 3,331 amino acid positions. The amino acid dataset comprised only the protein-coding genes.

Moreover, we also constructed a nuclear dataset comprising only 18S, 28S and EF-1α sequences at the nucleotide level for these 17 taxa (Table 1). The nuclear dataset comprised 5,526 nucleotide positions. Analyses of nuclear ribosomal gene datasets were also based on 32 and 61 taxa to reveal if taxon sampling had a substantial impact on the phylogenetic reconstruction of the nuclear data. By comparison, taxon sampling was far more limited for mitochondrial genome sequences. Additional File 2 provides a summary of the construction of these datasets with more than 17 taxa.

### Phylogenetic Analyses

Maximum likelihood (ML) and Bayesian inference (BI) approaches were employed for all mitochondrial, nuclear and combined datasets. For all nucleotide datasets with 17 taxa, ML analyses were performed in PAUP4.0b10 [32] with a GTR+Γ+I model as determined by Modeltest v3.7 based on the Akaike information criterion (AIC) [33,34]. Heuristic searches were run with random-taxon addition (10 replicates) using Tree-Bisection-Reconnection (TBR) swapping. All model parameters used fixed values as determined by Modeltest v3.7. Bootstrap analyses employed 1,000 iterations using heuristic searches with 10 random taxa addition replicates. Partitioned ML analyses were conducted with RAxML 7.2.8 [35] using a GTR+Γ+I model for each individual gene and 200 bootstrap replicates followed by a best tree search. Partitioned BI invoked independent substitution models for each gene in MrBayes version 3.1.2 [36] and ran for 5*10^6 ^(mitochondrial and nuclear) or 2*10^6 ^(combined) generations, respectively, with 2 runs of 4 chains (3 heated and 1 cold). Trees were sampled every 100 generations. The implemented diagnosis feature comparing the 2 runs by average standard deviation of split frequencies was determined every 10,000 generations. GTR+Γ+I models were selected under the AIC in MrModeltest [37,38] for 18S and 28S rDNA, EF-1α, *cox1*, *cox2*, *cob*, *nad1*, *nad3*, and *nad4*, GTR+I models for both 12S and 16S rDNA, GTR+Γ model for *cox3*, and HKY+Γ model for *nad2, nad4L *and *nad5*. Convergence of -ln likelihood scores and tree length was determined using Tracer v1.4.1 [39] to identify the *burnin *point at which all estimated parameters reached equilibrium (*burnin *= 100 trees). The majority-rule consensus tree containing posterior probabilities (PP) was determined from the remaining trees. Additional File 2 provides a more detailed description of the analyses and results for the datasets with more than 17 taxa.

For both amino acid datasets (mitochondrial and combined data with 17 taxa), non-partitioned and partitioned ML, and partitioned BI analyses were run. For ML analyses, model selection was performed in RAxML 7.2.8 [35] and the MtZOA+Γ+I+F model was chosen as the best-fitting one for both non-partitioned datasets. For individual genes, MtZOA+Γ+I models were selected for *cox1*, *cox2 *(additionally +F), *cox3 *and *cob*, and DAYHOFF+Γ+I for *nad1*, *nad2, nad3*, *nad4*, *nad4L*, *nad5 *and EF-1α. Maximum likelihood searches were implemented with 200 bootstrap replicates using RAxML [35] followed by a ML tree search for both non-partitioned and partitioned ML analyses. For partitioned BI of amino acid datasets, the mixed amino acid substitution model option plus a Γ distribution and a proportion of invariant sites was assigned to each partition individually and unlinked in MrBayes v3.1.2. BI ran for 2*10^6 ^generations and trees sampled every 500 generations (*burnin *= 20 trees). In the mixed model option, a specific model is not specified *a priori*, but each model is chosen during the run based on its posterior probability.

#### Non-stationary sequence evolution

To analyze data in a non-stationary Bayesian framework, we used PHASE 2.0 [40] to allow usage of different compositional vectors along branches of the tree. As in stationary Bayesian inferences using MrBayes, we conducted partitioned analyses for nucleotide datasets with 17 taxa of both mitochondrial and nuclear data invoking previously mentioned substitution models for each gene (except that the proportion-of-invariant-sites parameter is not available in PHASE 2.0). We performed analyses based on 3, 6 or 9 different compositional vectors. For each number of compositional vectors, we ran 4 independent runs, with one cold chain each and different random seeds (i.e., 3, 11, 88, and 1000), in parallel. Each run ran for 12*10^6 ^generations and trees were sampled every 1,000 generations. The first 2*10^6 ^generations were discarded as *burnin *as convergence of -ln likelihood scores and tree length was indicated by Tracer v1.4.1[39].

#### Topology testing

To further understand congruence and incongruence in our datasets, the Approximately Unbiased (AU) topology test of CONSEL [41,42] was employed to assess support for alternative hypotheses. More specifically under the ML criterion, AU tests compared the three possible terebelliform hypotheses with respect to incongruence for each possible combination of partitions in the 17-taxa case (i.e., 18S, 28S, mtDNA, 18S/28S, 18S/EF-1α, 18S/mtDNA, 28S/EF-1α, 28S/mtDNA, EF-1α/mtDNA, 18S/28S/EF-1α, 18S/28S/mtDNA, 18S/EF-1α/mtDNA, 28S/EF-1α/mtDNA, and 18S/28S/EF-1α/mtDNA). Based on initial results, the following hypotheses were tested: 1) Trichobranchidae as sister to Alvinellidae/Ampharetidae (TriAA), 2) Trichobranchidae as sister to Terebellidae (TriTer), and 3) Terebellidae as sister to Alvinellidae/Ampharetidae (TerAA). PAUP analyses were constrained to obtain only the best trees congruent with the particular hypothesis. Settings for the analyses were as described above.

### Spectral Analyses

We conducted spectral analyses to gain further insights into the support for specific bipartitions (or splits) [43,44] because they have been useful in the detection of the symplesiomorphy trap [17]. A bipartition splits a set of OTUs into two groups. In the context of spectral analyses, we use the term *ingroup *(italicized here to distinguish its usage in spectral analyses from common systematic usage) to define the group of the bipartition we are interested in, and *outgroup *for the other group of that bipartition. For example, Trichobranchidae, Alvinellidae and Ampharetidae in one group of the bipartition, the *ingroup*, and all others including Terebellidae in the other, the *outgroup*, would be congruent with the TriAA hypothesis. To calculate and visualize the bipartition support, we used Splits Analyses MethodS [SAMS, [17]] and Microsoft Excel for mitochondrial, nuclear and combined datasets with 17 taxa. SAMS is a split-decomposition tool that does not require Hadamard conjugations. Hence, there is no need to consider the complete split space. SAMS differentiates support for a bipartition into three categories: 1) binary, both groups exhibit only one character state each, but different from each other; 2) noisy *outgroup *(i.e., while the *ingroup *exhibits only one state the *outgroup *exhibits more than one state, though a majority state within the group can still be identified); 3) noisy *ingroup *and *outgroup *[17]. Because we were only interested in bipartitions regarding relationships within Terebelliformia, we only retrieved bipartitions from the results that were relevant regarding these relationships. The PERL script to retrieve these bipartitions is available from THS upon request.

### Determination of Compositional Biases

We also analyzed our nuclear and mitochondrial datasets for compositional biases, which can mislead phylogenetic analyses [e.g., [15,45-53]]. First, we employed relative composition variability (RCV), which is the average variability in composition between taxa for a dataset [15]. Phillips and Penny [15] used absolute numbers of nucleotide occurrence for calculation of RCV. However, this means that the RCV value does not only reflect composition variability, but also sequence length variability in the dataset. Therefore, we created a measure of relative composition frequency variability (RCFV) by modifying the RCV calculation to use base frequencies instead of absolute numbers:

$$RCFV=\overset{n}{\underset{i=1}{\sum }}\frac{\left|\mu_{Ai}-{\tilde{\mu }}_{A}|+|\mu_{Ci}-{\tilde{\mu }}_{C}|+|\mu_{Gi}-{\tilde{\mu }}_{G}|+|\mu_{Ti}-{\tilde{\mu }}_{T}\right|}{n}$$

where μ_A*i *_is the base frequency of A for the *i*th taxon and ${\tilde{\mu }}_{A}$ is the mean base frequency across *n *taxa. Besides the RCFV for complete datasets, we also report herein taxon-specific RCFV values $\left(\text{i}\text{.e}.,\left(\left|\mu_{Ai}-{\tilde{\mu }}_{A}|+|\mu_{Ci}-{\tilde{\mu }}_{C}|+|\mu_{Gi}-{\tilde{\mu }}_{G}|+|\mu_{Ti}-{\tilde{\mu }}_{T}\right|\right)∕n\right)$, taxon-specific absolute deviations of each nucleotide $\left(\text{e}.\text{g}.,|\mu_{Ai}-{\tilde{\mu }}_{A}|\right)$, and combinations of nucleotides (i.e. AT or GC and Y or R). Second, we determined different skew values to determine if strong biases between two nucleotide frequencies exist. Perna and Kocher [54] introduced the A-T and G-C skews for an individual strand of nucleic acids. Herein, we additionally propose A-G and C-T skews, because for mitochondrial genomes, major mutational biases are within purine and pyrimidine frequencies, respectively [55]. A-G and C-T skews for a taxon are calculated the same way as A-T and G-C skews are:

$$A-G\;skew=\frac{\mu_{A}-\mu_{G}}{\mu_{A}+\mu_{G}};C-T\;skew=\frac{\mu_{C}-\mu_{T}}{\mu_{C}+\mu_{T}}$$

## Results

### Phylogenetic Analyses

#### Mitochondrial datasets

**Figure 2:**
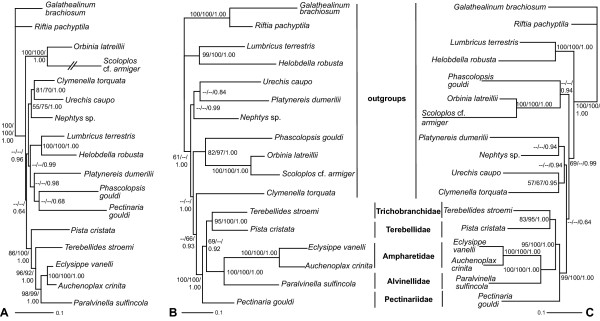
**Phylogenetic reconstructions using nuclear, mitochondrial and combined nucleotide datasets with 17 taxa**. (A) Nuclear ML tree. The branch leading to *Scoloplos *cf. *armiger *was reduced by 75%. (B) Mitochondrial ML tree. (C) Combined ML tree. All trees represent identical topologies regarding terebelliform relationships for both ML and partitioned BI. Nodal support values are given at branches in the order: non-partitioned ML bootstrap, partitioned ML bootstrap and PP of the BI. A dash indicates < 50%.

ML and partitioned BI analyses of 17-taxa mitochondrial datasets based on either nucleotides or amino acids inferred identical topologies, with one exception, regarding terebelliform relationships with strong nodal support (Figure 2b & Additional File 3). Monophyly of Terebelliformia is well supported (BS: 100 for non-partitioned nucleotide (nNuc) and partitioned nucleotide (pNuc) analyses, 93 for non-partitioned amino acid (nAA), and 94 for the partitioned amino acid (pAA) analyses; PP: 1.00 for both BI analyses). Mitochondrial datasets infer a sister relationship between Trichobranchidae and Terebellidae, the TriTer hypothesis (BS: 95 for nNuc, 100 for pNuc, 62 for nAA and 84 for pAA; PP: 1.00 for both). Furthermore, topology testing significantly rejected a sistergroup relationship of Trichobranchidae to Alvinellidae/Ampharetidae, the TriAA hypothesis (p = 0.003), as well as Terebellidae as sister to Alvinellidae/Ampharetidae, the TerAA hypothesis (p = 0.028). Two Ampharetidae taxa were close to Alvinellidae in the analyses of both mitochondrial datasets (BS: 100 for all four; PP: 1.00 for both). Pectinariidae was shown to be the basal lineage in Terebelliformia except in the partitioned ML analysis of the nucleotide dataset, which placed Pectinaridae as sister to Trichobranchidae/Terebellidae (BS: 72, data not shown).

#### Nuclear datasets

ML and partitioned BI of the 17-taxa, three-nuclear-gene (i.e., 18S, 28S and EF-1α) dataset inferred an identical topology with respect to terebelliform relationships (Figure 2a). Interestingly, monophyly of Terebelliformia was not recovered as *Pectinaria gouldi *was placed as sister to the sipunculid *Phascolopsis gouldi*, albeit with weak support (Figure 2a). The other four terebelliform taxa formed a clade with stronger nodal support (BS: 86 for nNuc, 100 for pNuc; PP 1.00) than in mitochondrial analyses (BS: 69 for nNuc, <50 for pNuc; PP: 0.92, Figure 2b). As for the mitochondrial analyses, a sistergroup relationship of Alvinellidae and Ampharetidae is well corroborated (BS: 98 for nNuc, 99 for pNuc; PP: 1.00). Moreover, the TriAA hypothesis was supported (BS: 96 for nNuc, 92 for pNuc; PP: 1.00) and topology testing significantly rejects the alternative TriTer (favored by the mitochondrial data) and TerAA hypotheses (p = 0.038 and p = 0.006, respectively).

#### Combined datasets

Phylogenetic trees from combined analyses (Figure 2c & Additional File 3) were similar to the ones from mitochondrial data (Figure 2b) with differences occurring in outgroup relationships. Monophyly of Terebelliformia is significantly supported in these analyses (BS: 99 for nNuc, 100 for pNuc, 98 for nAA and 93 for pAA; PP: 1.00 for both; Figure 2c, Additional File 3). Pectinariidae branched off first within terebelliforms (BS: 95 for nNuc, 100 for pNuc, 96 for nAA and 72 for pAA; PP: 1.00 for both). Alvinellidae was recovered as sister to Ampharetidae (BS: 100 for all four; PP: 1.00 for both). Trichobranchidae was placed as sister to Terebellidae, the TriTer hypothesis, in all analyses. However, bootstrap support for the TriTer hypothesis in the combined analyses was generally lower than in mtDNA alone analyses (83 in nNuc, 95 in pNuc, 41 in nAA, and 74 in pAA compared to 95, 100, 62, and 84, respectively; Figure 2 & Additional File 3). Furthermore in contrast to the mitochondrial Nuc dataset, topology testing did not significantly reject the alternative TriAA hypothesis favored by the nuclear dataset (p = 0.184), though the TerAA hypothesis is still significantly rejected (p = 0.012).

### Congruence and Incongruence between Partitions regarding Terebelliformia

**Figure 3:**
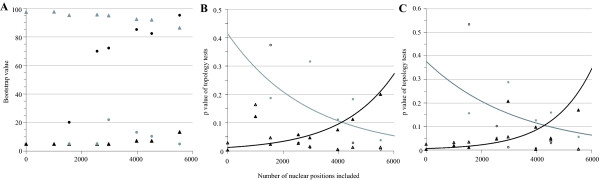
**Analyses evaluating incongruence of mitochondrial and nuclear data concerning placement of Trichobranchidae**. (A) Bootstrap support. (B) Results of the topology tests against the best tree. (C) Same as B, but with the long-branched taxa *Pectinaria gouldi*, *Phascolopsis gouldi *and *Scoloplos *cf. *armiger *excluded from the analyses. Black symbols indicate TriAA, grey symbols the TriTer and open symbols the TerAA hypothesis. Circles stand for all possible combinations of only the nuclear partitions and triangles for mitochondrial data plus all possible combinations of the nuclear partitions.

Due to these results, we further explored conflict regarding the TriTer and TriAA hypotheses indicated by mtDNA (Figure 2b) or nuclear partitions (Figure 2a), respectively. Therefore, we conducted phylogenetic analyses and topology testing for all possible combinations of the four partitions (18S, 28S, EF-1α, mtDNA) when using 17 taxa. These analyses showed that when the mitochondrial data partition was added, the TriTer hypothesis was supported, whereas all possible combinations of the three nuclear genes, excluding mtDNA data, recovered the TriAA hypothesis. With an increasing amount of nuclear data (mitochondrial partition excluded) bootstrap support for the TriAA hypothesis steadily increased (black circles in Figure 3a), while bootstrap support for the TriTer hypothesis remained low (grey circles in Figure 3a). Furthermore, the p value of the AU test for the TriTer hypothesis decreased with an increasing amount of nuclear data from a non-significant value of 0.447 to a significant one of 0.041 (Figure 3b, grey circles and trend line). On the other hand, in all datasets including mitochondrial data bootstrap support for the TriTer hypothesis was high, though it slightly decreased with an increasing amount of nuclear data (grey triangles in Figure 3a), and, vice versa, the bootstrap support for the TriAA hypothesis was low, but slightly increased with increasing nuclear data (black triangles in Figure 3a). However, as the proportion of nuclear data combined with mtDNA data increased, the p value of the AU test for the TriAA hypothesis became less significant (Figure 3b, black triangles and trend line; p values change from 0.004 to 0.184). Comparatively and independent of the inclusion of mitochondrial data, the p value for the TerAA hypothesis decreased with an increasing amount of nuclear data (open triangles and circles in Figure 3b). Hence, topology tests clearly revealed that nuclear data favor the TriAA hypothesis, whereas mitochondrial data favor the TriTer hypothesis.

### Spectral Analyses

**Figure 4:**
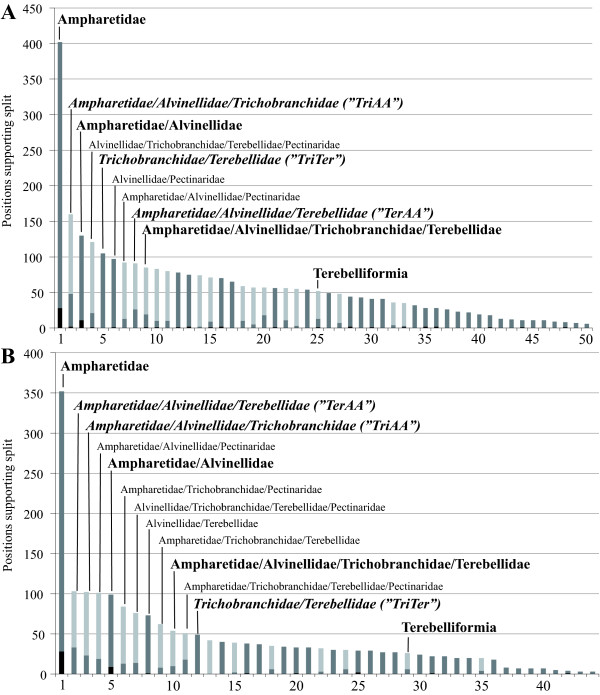
**Results of spectral analyses for all splits recovered by SAMS concerning terebelliform relationships**. (A) Nuclear and (B) mitochondrial datasets with 17 taxa. Only supporting positions for the *ingroup *of the split are shown and not the *outgroup*. Names in bold at splits were recovered in our analyses. Additionally, the TriAA, TriTer and TerAA hypotheses are in italic. Black indicates binary positions, that is both groups exhibit only one character state each, but different from each other; dark grey noisy *outgroup *positions, while the *ingroup *exhibits only one state, the *outgroup *exhibits more than one state, though a majority state within the group can still be identified; light grey noisy *ingroup *and *outgroup *positions supporting a split.

Spectral analyses revealed that 160 positions of the 17-taxon nuclear dataset support the TriAA hypothesis (Figure 4a) recovered in the best tree (Figure 2a). One hundred and five positions are consistent with the TriTer hypothesis favored by the mtDNA data and 91 with the TerAA hypothesis. This is congruent with the results of the topology tests based on the 17-taxon nuclear dataset, where the TriTer hypothesis had a higher p value than the TerAA hypothesis (0.038 > 0.006). However for the mitochondrial dataset with 17 taxa, similar numbers of positions, 103 and 102, support the TerAA and TriAA hypothesis, respectively. On the other hand, only 49 positions are consistent with the TriTer hypothesis, which was recovered by the best tree of the mitochondrial dataset (Figure 2b).

Besides the number of positions, the quality of supporting positions is different for these three alternative hypotheses in both 17-taxon datasets. For the nuclear dataset, two binary positions support the TriAA hypothesis (black color in Figure 4a) and no binary positions support the TriTer and TerAA hypotheses. In contrast, no binary positions are found to support any of the three hypotheses in the mitochondrial dataset. All other positions consistent with the TriAA or TerAA hypothesis are either noisy only in the *outgroup *(dark grey in Figure 4) or in both *ingroup *and *outgroup *(light grey in Figure 4), with more positions belonging to the latter class. Conversely, positions consistent with the TriTer hypothesis are exclusively based on a single class of positions, noisy in the *outgroup *only (Figure 4).

### Source of Incongruence

Based on analyses herein, placement of Trichobranchidae is incongruent between mitochondrial and nuclear data. To further investigate possible sources of incongruence with regards to Trichobranchidae placement, we examined two properties known to mislead placement of taxa, placement of the root and base composition heterogeneity.

#### Placement of the root

**Figure 5:**
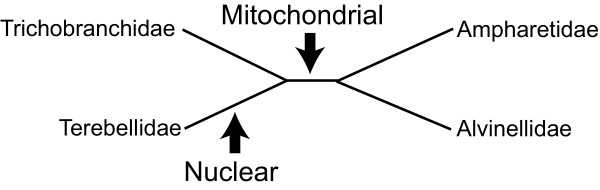
**The unrooted subtree of Trichobranchidae, Terebellidae, Alvinellida and Ampharetidae**. Arrows indicate the position of the root by either nuclear or mitochondrial data.

With respect to the relationships of Trichobranchidae, Terebellidae, Alvinellidae and Ampharetidae to each other, mitochondrial and nuclear partitions yield identical subtrees that were rooted differently (Figure 5). Effects of long-branched outgroups and basal taxa misleading placement of the root have been long known [for review see [56]]. *Pectinaria gouldi*, as well as *Phascolopsis gouldi*, exhibit long branches in nuclear rRNA data [[19,57] and see also Additional File 2]. However, Pectinariidae is placed as sister to the other terebelliform taxa and may influence placement of Trichobranchidae within the nuclear dataset (Figure 2, Additional File 2). Nuclear data of *Scoloplos *cf. *armiger *also exhibited a long branch on the reconstructed topology (Figure 2a). Therefore, we excluded these taxa (*Pectinaria gouldi*, *Phascolopsis gouldi*, *Scoloplos *cf. *armiger*) to examine the possibility of long-branch attraction, but found that they did not influence placement of the root or Trichobranchidae. All combinations of nuclear genes still favored the TriAA hypothesis, whereas the addition of the mitochondrial data always rendered Trichobranchidae being sister to Terebellidae in ML reconstructions. Correspondingly, results of topology tests are not altered substantially by excluding these three long branched taxa (compare Figure 3c with Figure 3b).

Poor taxon sampling can also influence taxon placement and rooting [58,59]. As we could not easily increase the available number of mitochondrial genomes for Terebelliformia, we focused on adding more nuclear data and included 18 new 18S and 13 28S sequences for Terebelliformia and one cirratulid to the available data (Additional File 2). Phylogenetic analyses of this dataset comprising 32 taxa also recovered a sistergroup relationship of Trichobranchidae to Alvinellidae/Ampharetidae (BS: 80; PP: 0.95) within a monophyletic Terebelliformia. Additionally, the 61-taxon dataset based only on 18S rRNA data failed to provide resolution within Terebelliformia (Additional File 2); thus, neither exclusion of long-branched taxa nor an increased taxon sampling had an influence on the placement of the root for the nuclear data.

#### Base composition

**Figure 6:**
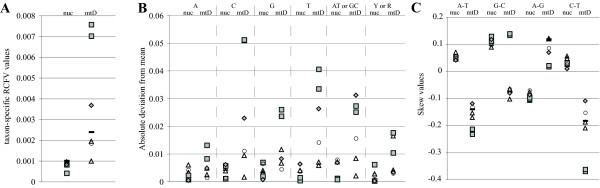
**Analyses of compositional heterogeneity in nuclear and mitochondrial datasets in the mitochondrial protein-coding genes**. (A) Taxon-specific relative composition frequency variability (RCFV). (B) Absolute deviation from mean frequency for adenine (A), cytosine (C), guanine (G), and thymine (T) as well as combinations of adenine/thymine (AT) or guanine/cytosine (GC) and of pyrimidines (Y) or purines (R). Only one absolute value is provided for AT and GC or Y and R as only two character states are now present and any change in one state has the exact opposite negative or positive value in the other. (C) Skew values within the combinations adenine/thymine (A-T), guanine/cytosine (G-C), purines (A-G) and pyrimidines (C-T). Ampharetidae (grey squares), Alvinellidae (grey diamonds), Pectinariidae (open circles), Trichobranchidae and Terebellidae (both open triangles), mean values of outgroup taxa (black bar), nuclear (nuc), mitochondrial (mtD) .

Evaluations of base composition heterogeneity showed a strong difference between nuclear and mitochondrial data. The RCFV value for mitochondrial data (0.0494) was much greater than for nuclear data (0.0159). Thus, mitochondrial data exhibit a stronger compositional heterogeneity. For mitochondrial data, taxon-specific RCFV values (Figure 6a) showed that Alvinellidae, and especially Ampharetidae, had much higher values than the other terebelliforms or the average outgroup value indicating strong compositional biases in Alvinellidae and Ampharetidae. No obvious biases were observed in nuclear data. Similar results were obtained for absolute deviations from mean frequency for individual nucleotides as well as combinations of nucleotides (Figure 6b). For pyrimidines (cytosine and thymine), Ampharetidae and Alvinellidae deviated more from the mean than other terebelliform taxa. In addition, Ampharetidae also showed a much stronger deviation from the mean in guanine. Binning nucleotides as AT and GC did not alleviate these differences in deviation (and even made it more pronounced for Alvinellidae), but recoding pyrimidines (Y) and purines (R) reduced the biases between terebelliform taxa (Figure 6b).

Ampharetidae exhibited a strong G-C skew value towards guanine relative to cytosine (Figure 6c). Moreover for mitochondrial data, C-T skews indicated that Ampharetidae was biased towards thymine, and Alvinellidae away from it, relative to other taxa. The same pattern could be observed in A-T skews driven by the differences in thymine frequencies. Thus, Ampharetidae and Alvinellidae showed strong-but opposite-biases in frequencies of pyrimidines, and Ampharetidae also a strong skew towards guanine. These evaluations were based on the mitochondrial dataset, we used for phylogenetic analyses (i.e., excluding 3^rd ^positions), but examining either 3^rd ^positions alone or with 3^rd ^positions included resulted in similar patterns (Additional File 4). Codon usage reflected biases in base frequencies with deviations in Ampharetidae and Alvinellidae compared to the other taxa (Additional File 1).

### Amelioration of Incongruence

#### Non-stationary sequence evolution

Using models of non-stationary sequence evolution has successfully ameliorated misleading effects of compositional biases in mitochondrial genomes of beetles [60]. Therefore, we also employed such models for both our mitochondrial and nuclear datasets using PHASE 2.0 [40]. For both datasets and each number of different compositional vectors, 4 independent chains starting from different random seeds failed to converge upon the same score indicating a structured tree-space with several local optima. Nonetheless for mitochondrial data, the majority-rule consensus topology derived from the best run (i.e, -lnL values) for each number of different compositional vectors (i.e., 3, 6, or 9) were identical except for the position of the outgroup taxon *Clymenella torquata *(Additional File 5). As before with mitochondrial data, Terebellidae and Trichobranchidae were sister to each other (PP: 1.00 for all three; Additional File 5). For nuclear data, the three topologies derived from the best runs invoking 3, 6 or 9 different vectors placed Trichobranchidae as sister to Alvinellidae/Ampharetidae (PP: 1.00 for all three; Additional File 5). Thus, using different compositional vectors along the branches did not reduce incongruence between datasets.

#### RY coding

**Figure 7:**
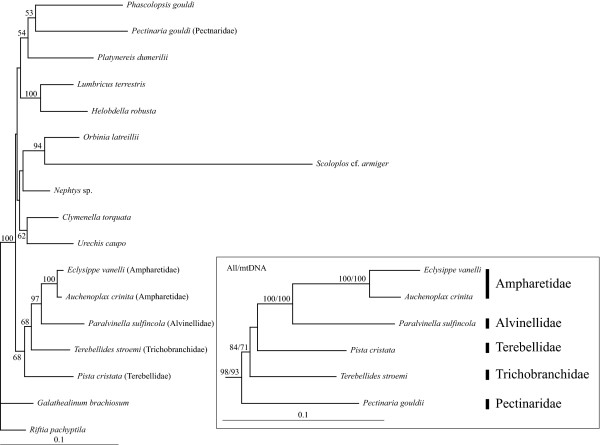
**Phylogenetic reconstructions using nuclear, mitochondrial and combined datasets based on RY coding**. Only the nuclear ML tree is completely shown. With respect to terebelliform relationships, analyses of the mitochondrial and combined dataset recovered the same topology. Therefore, in the inlet only this part of the mitochondrial ML tree is shown and no outgroups. Only bootstrap values above 50 are shown. In the inlet, bootstrap values of the mitochondrial analysis are given at the first position and of the combined analysis at the second.

For mitochondrial genomes, RY coding strategies can ameliorate biases within pyrimidines and purines, because they do not distinguish between transition or transversion classes [15,61]. The best ML tree based on RY coding of the nuclear partition (Figure 7) is similar to the ML tree using standard nucleotide coding (Figure 2a; with the exception of *Scoloplos *cf. *armiger*/*Orbinia latreillii *placement). However, bootstrap support for Trichobranchidae as sister to Alvinellidae/Ampharetidae dropped.

In contrast, RY coding of the mitochondrial partition and combined dataset (inset in Figure 7) yielded different ingroup relationships (see Figures 2b &2c for standard nucleotide coding) with Terebellidae as sister to Ampharetidae/Alvinellidae rather than Trichobranchidae. Notably, bootstrap support for this clade was below 50 in the analyses of both mitochondrial and combined data and all previous topology tests clearly rejected this relationship (Figures 3b &3c). Besides this difference in ingroup relationships, RY coding of mitochondrial and combined data also differed in several outgroup relationships.

## Discussion

Biases in nucleotide frequencies influenced placement of Trichobranchidae and Terebellidae in both mitochondrial and combined analyses. Misplacement of these taxa is interesting because the taxa themselves did not exhibit compositional biases, but Alvinellidae and Ampharetidae biases influenced their placement. This misplacement was apparently due to biases in Ampharetidae and Alvinellidae and can be related to the "symplesiomorphy trap" for which few molecular examples have been elucidated [16,17]. In the Cirripedia example by Wägele and Mayer [17] (Figure 1B), Acrothoracica and Ascothoracida grouped together due to symplesiomorphic characters because of the long branch uniting the remaining Cirripedia. Though no long branches could be observed in our analyses based on mitochondrial data regarding terebelliform taxa, biases in base composition and codon usage detected in Ampharetidae and Alvinellidae pointing in opposite directions appear to have had a similar effect. These directional biases affected nucleotides in all three coding positions of mitochondrial genes in Ampharetidae and Alvinellidae presumably due to differences in substitution rate or pattern.

In our case the symplesiomorphy trap appears to have misrooted a terebelliform subtree rendering a paraphyletic assemblage as a monophyletic group. The misinterpretation appears due to basal homologies, or symplesiomorphies, rather than an artificial signal due to homoplasy (e.g., long branches). First of all, though Alvinellidae and Ampharetidae are affected by opposite biases in mitochondrial nucleotide frequencies their sistergroup relationship, which is independently confirmed by the nuclear data, is still strongly supported by mitochondrial data as judged by bootstrap and spectral analyses. Hence, these two taxa appear unaffected by the opposite biases. Second, we could exclude that the nuclear partition is affected by an artificial signal; the nuclear data exhibited no biases with respect to terebelliform taxa. The root of the subtree comprising Terebellidae, Trichobranchidae and Ampharetidae/Alvinellidae, which was supported by all our analyses as well as several previous ones [e.g., [19,57,62]], was not placed differently by the inclusion or exclusion of taxa [56]. Moreover, the spectral analysis of the nuclear partition is in agreement with the reconstructed nodes regarding the relations of these three taxa to each other. The number of supporting positions in the spectral analysis is in agreement with support by bootstrap and topology test p values for nuclear data. Third and contrasting with the nuclear data, the spectral analyses of the mitochondrial data are not congruent with tree reconstructions. Whereas the TriTer hypothesis was recovered in all best trees that included mtDNA data and was strongly supported by bootstrap and topology test results, spectral analyses revealed that this hypothesis was consistent with the fewest numbers of positions in the mitochondrial data. Using mitochondrial data, these characters overwhelmed the larger numbers of positions supporting the alternative placement of Trichobranchidae.

**Figure 8:**
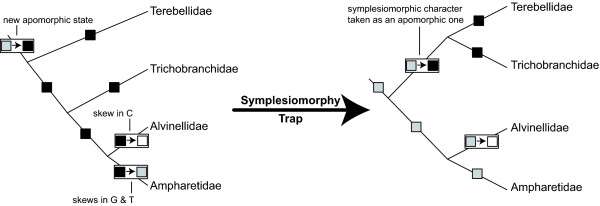
**Schematic representation of the effect of biases with respect to the symplesiomorphy trap in our terebelliform example**. White, grey and black boxes indicate different character states as well as the possible change of one state to another along a branch.

In the case of the symplesiomorphy trap, the phylogenetic signal for a certain relationship can be eroded along internal branches leading to subgroups without affecting the subgroups themselves. In the Cirripedia example [17], this erosion occurred along the branch leading to all Cirripedia but Acrothoracica (Figure 1B). In our case, there are more possibilities; the branch leading to Ampharetidae/Alvinellidae as well as the branches within this clade could be relevant. For the Terebellidae/Trichobranchidae/Ampharetidae/Alvinellidae clade, differences in substitution processes of Alvinellidae and Ampharetidae obscured signal for this clade by exhibiting a state different from the apomorphic state of this clade in one or both of these two taxa (Figure 8). Hence, a large proportion of the data would still exhibit the original character-state only in Terebellidae and Trichobranchidae, but not in Ampharetidae/Alvinellidae. As only four character states are exhibited in nucleotide data and because of skews in mitochondrial nucleotide frequencies, the likelihood is high that, in this case, states exhibited in Ampharetidae, Alvinellidae, or both, are also present in either Terebellidae or Pectinaridae. Accordingly, results of spectral analyses showed that 1) most of the positions in mitochondrial data supporting the split of Trichobranchidae/Ampharetidae/Alvinellidae are noisy within *ingroup *and *outgroup*, and 2) equal in numbers to the splits of Terebellidae/Ampharetidae/Alvinellidae and Pectinaridae/Ampharetidae/Alvinellidae (Figure 4b). Therefore, as with the Cirripedia example, strong support for the sistergroup relationship of Terebellidae and Trichobranchidae by mitochondrial data is due to symplesiomorphic characters rather than apomorphic ones.

The process of deamination of the non-coding strand may be responsible for biases observed herein for pyrimidines and purines [55]. Compositional biases in our mitochondrial data were greater within pyrimidines than in purines; guanine had the lowest average frequency (16%) of all nucleotides. This is similar to the situation found in mammals though their guanine frequency can be considerably lower [15,55,63,64]. In mammals, this is due to spontaneous deamination of cytosine to uracil and adenine to hypoxanthine on the complementary strand during replication of mitochondrial genomes [55]. The former deamination occurs more often than the latter [65] explaining the low level of guanines in mammals on the coding strand and the stronger bias observed in pyrimidines than in purines, because the low guanine frequency allows for little variation [15].

The best strategy to ameliorate the effect of the symplesiomorphy trap is to increase ingroup taxon sampling [17]. However, increasing the taxon sampling might not always be easily achieved or possible. For example, sampling of nearly complete mitochondrial genomes in annelids is time consuming and expensive, but new sequencing technologies are changing this. In other cases, taxon sampling will be limited by number of extant taxa from which genetic material can be obtained. Therefore, we tested different strategies with respect to their capabilities to ameliorate the effect of the symplesiomorphy trap given a limited taxon sampling. In the Cirripedia example, using appropriate methods such as ML and increased outgroup sampling ameliorated the symplesiomorphy problem because this misplacement was due to long branches [66]. In the Mammalia example, the problem could be solved by the RY coding strategy and partitioned analyses, which resulted in weak support for the Theria hypothesis even using mitochondrial data [15]. Moreover, usage of non-stationary models of sequence evolution were able to adjust for compositional biases in mitochondrial genomes in the reconstruction of the beetle phylogeny [60].

In our case, the most effective strategy was RY coding, which reduced the effects of compositional biases within pyrimidines and purines. However, we still did not recover strong support for Trichobranchidae as sister to Ampharetidae/Alvinellidae with either mitochondrial or combined data. Moreover, phylogenetic signal in all datasets was substantially decreased by RY coding. Addition of nuclear data was only able to slightly minimize the effects of the symplesiomorphy trap as indicated, for example, by the slight decrease in bootstrap support for the presumed 'incorrect' hypothesis. Therefore, substantially more unbiased nuclear data would have been necessary to turn the tides. On the other hand, herein partitioned analyses always obtained the same topology as non-partitioned ML analyses, and PHASE analyses did not resolve incongruence either. The poor performance of non-stationary models of sequence evolution in our analyses, in comparison to Sheffield et al. [60], might be due to the limited sampling of ingroup taxa. Increased sampling may allow better adjustment to biases along the branches [58,59]. Finally, we also tested if exclusion of biased taxa in turn would alter the results [56], but there was no noticeable effect. Thus, though several approaches were tried, none completely ameliorated the influence of the symplesiomorphy trap.

Interestingly, results based on combined data seem to be congruent with morphological and mitochondrial gene order data and, therefore, the underlying incongruence in the data was not apparent at first. Trichobranchidae strongly resemble Terebellidae and, thus, were placed as sister to or within Terebellidae [18,20,67]. However, only one non-homoplastic character supports their common origin: prostomium on peristomium with fused frontal edges. In contrast, others did not support a sister relationship of Terebellidae and Trichobranchidae [68,69]. The position of two adjacent *trnM *genes also seemed to support such a relationship of Terebellidae and Trichobranchidae [23]. However, two adjacent *trnM *genes are also found in the pectinarid *P. gouldi *(Additional File 1) and in some but not all sipunculids [70-72]. Thus, no unequivocal character supports a sistergroup relationship of Terebellidae and Trichobranchidae. Analyses herein revealed that support by mitochondrial and combined data was only due to symplesiomorphic characters. On the other hand, although a close relationship between alvinellids and ampharetids has been long suspected based on morphology [e.g., [18,69,73]], until now strong support by molecular data [e.g., [19,68]] has been lacking.

## Conclusions

Herein we report the detection of the symplesiomorphy trap in molecular data, one of a few known examples to date. Mitochondrial data placed Trichobranchidae as sister to Terebellidae in contrast to the nuclear data, which placed Trichobranchidae as sister to Ampharetidae and Alvinellidae. These latter two taxa exhibited strong compositional biases in the mitochondrial data as shown by spectral analyses as well as skew and RCFV values. However, Ampharetidae and Alvinellidae themselves were not misplaced but caused Trichobranchidae to be erroneously placed. This taxon exhibits no obvious compositional bias. Unfortunately, several state-of-the-art approaches (i.e., partitioning the dataset, performing ML analyses and partitioned analyses, use of several outgroup taxa, exclusion of biased taxa, use of different numbers of compositional vectors to implement time-heterogeneous models) were not able to ameliorate the influence of the symplesiomorphy trap in the mitochondrial data. Therefore, more sophisticated substitution models have to be developed to appropriately address this peculiar tree reconstruction artifact. In the mean time, partitioned and careful analyses can be used to detect the trap and to be aware of incongruencies in the molecular data even if nodal support is high as in our case. Given the advent of next generation sequencing technologies, we hope that analyses, such as those done here, will be better able to detect artifacts due to systematic errors because much more data will be brought to bear on such issues. Hence, these approaches may add strength and confidence to results of phylogenomic studies by allowing more in depth understanding of the sources of signal and noise.

## Competing interests

The authors declare that they have no competing interests.

## Authors' contributions

THS and KHM conceived this study. BH, AG and MN collected the nuclear data and MZ the mitochondrial data. THS and MZ performed the analyses. THS, MZ and KHM mainly contributed to writing the manuscript. All authors read and approved the final manuscript.

## Supplementary Material

Additional file 1 **Mitochondrial genomes and their properties**. This file provides a more detailed description of methods for the determination of the mitochondrial genomes as well as of their general properties such as codon usage. Click here for file

Additional file 2 **Analyses with increased taxon sets**. This file provides a summary of datasets, analyses and results with more than 17 taxa. Click here for file

Additional file 3 **Best ML trees of the amino acid datasets with 17 taxa**. This file provides a supplementary figure showing the best tree of ML and BI analyses based on mitochondrial and combined amino acid datasets. Click here for file

Additional file 4 **Compositional heterogeneity of the 3^rd ^positions**. This file provides a supplementary figure showing the analyses of compositional heterogeneity of 3^rd ^positions included in the mitochondrial dataset as well as of only the 3^rd ^positions of the mitochondrial protein-coding genes. Click here for file

Additional file 5 **Analyses using time-heterogeneous models**. This file provides a supplementary figure showing the results of the PHASE analyses using 3, 6 or 9 compositional vectors, respectively, for both the mitochondrial and nuclear dataset. Click here for file
