# Supplementary material for: Detecting the symplesiomorphy trap: a multigene phylogenetic analysis of terebelliform annelids
Source: BMC Evol Biol. 2011 Dec 20;11:369. doi: 10.1186/1471-2148-11-369 (PMC3282830; doi:10.1186/1471-2148-11-369)
Supplement: Additional file 1 — Mitochondrial genomes and their properties. This file provides a more detailed description of methods for the determination of the mitochondrial genomes as well as of their general properties such as codon usage. [file 1471-2148-11-369-S1.PDF]

**Online Appendix 1.** More detailed description of methods for the determination of the mitochondrial genomes and their properties like codon usage.

*mtDNA Data Collection*

*Auchenoplax crinita* and *Paralvinella sulfincola* – Mitochondrial genomes were amplified in four overlapping segments using species-specific primers. To generate these primers, conserved regions of *mLSU*, *cox1*, *cob* and *nad5* genes were amplified using taxonomically inclusive primers (Zhong et al. 2008), purified by QIAquick PCR purification kit (Qiagen), and products were sequenced in a CEQ8000 (Beckmann). From these data, four sets of species-specific primers for long PCRs were designed (Supplementary Table 1). HotStart long PCRs were performed on Eppendorf Mastercycler (Eppendorf) using Takara LA-Taq and the following protocol: 94 °C for 3min; then addition of polymerase followed by 35 cycles with 94°C for 30sec, 52°C for 30sec, and 70°C for 8min; final extension at 72°C for 10min and hold at 6°C. 50 µl long PCR reactions were set up as follows: 5µl 10×buffer, 8µl dNTP (2mM), 5µl $MgCl_2$ (25mM), 2µl of each long PCR specific primers (10µM each), 0.5µl Takara LA-Taq (5U/µl), 2µl DNA template and 25.5µl sterilized, distilled water. Both the *mLSU-cox1* and *cox1-cob* regions were about 4 kb in size. Both the *cob-nad5* fragment of *A. crinita* and the *nad5- mLSU* fragment of *P. sulfincola* were about 6-7 kb in size. The *nad5-mLSU* fragment of *A. crinita* and *cob-nad5* of *P. sulfincola* containing the UNK region were difficult to amplify consistent with previous reports for annelids (Boore and Brown 2000, Jennings and Halanych 2005, Zhong et al. 2008).

*Pectinaria gouldi* – Genome of *P. gouldi* was amplified in six segments. Conserved fragments of *mLSU*, *cox1*, *nad5* and *nad4* genes were amplified by taxonomically inclusive primers (Zhong et al. 2008) and sequenced. New primers were designed based on conserved regions in *cox3* (see Supplementary Table 1). Then specific long-PCR primers (Supplementary Table 1) were designed for amplification of *mLSU-cox1*, *cox1-cox3, nad5-nad4* and *nad4-mLSU*. The "universal" *cob* reverse primer "Cytb 876R" was used to pair with the *cox3* forward one to amplify *cox3-cob* fragment. Based on the sequence of this long fragment a specific *cob* forward long-PCR primer was designed to pair with the *nad5* reverse one to amplify the *cob-nad5* fragment. Long PCRs employed Takara LA-Taq as described above. The protocols of long PCR and purification were similar to above with some modifications of annealing temperatures based on different pairs of primers (Supplementary Table 1). All long fragments were sequenced directly by primers walking. We were unable to obtain a *cob-nad5* fragment, which presumably contains the UNK region. Information for all

sequencing primers for the three species can be found in the Supplementary Table 2.

*Mitochondrial gene order*

All three mitochondrial genomes (mtDNA) are uncompleted due to difficulty amplifying the UNK region. The partial mtDNA genome of *Auchenoplax crinita* (Ampharetidae) was 13,759 bp in length, of *Paralvinella sulfincola* (Alvinellidae) 13,640 bp, and of *Pectinaria gouldi* (Pectinaridae) 13,438 bp. From *A. crinita* we recovered 34 genes including 13 protein-coding genes, two rRNA genes and 19 tRNAs. There were 33 genes recovered in the *P. sulfincola* mtDNA including 12 protein-coding genes, two rRNA genes and 19 tRNAs, whereas the recovered *P. gouldi* genome contained 34 genes including 12 protein-coding genes, two rRNA genes and 20 tRNAs. Interestingly, the *P. gouldi* fragment possesses identical mitochondrial gene order as both the trichobranchid *Terebellides stroemi* and the terebellid *Pista cristata*. *P. gouldi* even possesses two adjacent copies of the methionine tRNA gene as found in the trichobranchid and terebellid (Zhong et al. 2008). Supplementary Figure 1 shows gene orders of all six available terebelliform mitochondrial genomes. All mitochondrial genes are transcribed from the same strand, similar to other annelids (Bleidorn et al. 2006a, Bleidorn et al. 2006b, Boore and Brown 2000, Boore and Staton 2002, Jennings and Halanych 2005, Zhong et al. 2008). Gene order of *A. crinita* is identical to the another published ampharetid *Eclysippe vanelli* except for the *trnF* between the *nad1* and *nad3* genes in *E. vanelli* (Zhong et al. 2008) that is presumably in the uncovered region of *A. crinita*.

Two adjacent *trnM* genes were discovered in the pectinariid *P. gouldi* as well as in terebellid and trichobranchid worms (Zhong et al. 2008), but not in an alvinellid and ampharetids. A unique duplication event was speculated based on their similar secondary structure, identical anticodons and the same sequences in stems. As Pectinariidae was highly supported as a basal clade of Terebelliformia, *trnM* duplication was noted to be a symplesiomorphy of terebelliforms instead of a synapomorphy for Trichobranchidae and Terebellidae (Zhong et al. 2008). Both Ampharetidae and Alvinellidae have secondarily lost one of the two *trnMs*. Besides, two adjacent *trnMs* were recently found in a complete mitochondrial genome of the sipunculid *Phascolosama esculenta* (Shen et al. 2009). Very similar sequences in the D and anticodon arms between the two *trnM*s indicate they may be from a duplication event, like in our study. However, such a duplication event has not been found in the other two published mitochondrial genomes of sipunculids, either a complete one of *Sipunculus nudus* (Mwinyi et al. 2009) or a partial one of *Phascolopsis gouldi* (Boore and Staton 2002), which covers this region. Comparing the mtDNA gene order across the

three sipunculid lineages, an identical protein-coding and rRNA gene order is evident. Considering for both *Sipunculus* and *Phascolopsis*, that their gene orders are very similar with exception of one *trnE* translocation, it cannot be expected that an extra *trnM* exists in the unobserved region of the *Phascolopsis* mitochondrial genome. The phylogenetic references of sipunculids suggest that the basal lineage *Sipunculus* is sister to the other two (Maxmen et al. 2003, Schulze et al. 2005), indicating that the *trnM* duplication in the *Phascolosama* lineage evolved potentially independently in Sipuncula from the one in Terebelliformia.

*Codon usage*

We also determined codon usage biases for the individual taxa using the average relative synonymous codon usage (RSCU). The RSCU of the protein-coding genes in all included annelids were calculated using the DNASTAR™ Lasergene program GENEQUEST (Burland 2000) (Supplementary Tables 3 & 4). We differentiated between degree of degeneration (4-fold or 2-fold), 3rd position nucleotides, and the location of corresponding tRNA (mitochondrial or nuclear genome). Thus, we had a total of ten classes: 4-fold degenerate NNA (mitochondrial), NNC, NNG, NNU (nuclear) and 2-fold degenerate NNA (mitochondrial), NNG (nuclear), NNA (nuclear), NNG (mitochondrial), NNC (mitochondrial), NNT (nuclear).

Ampharetidae used codons coding for 4-fold degenerated triplets less often than the other terebelliform taxa when codons possessed an adenine or cytosine at the 3rd position (Supplementary Fig. 2). The same was true for 2-fold degenerated codons ending on a cytosine. tRNAs of nuclear genomic origin with codons ending on guanine as well as 2-fold degenerated codons ending on uracil were used more often. Alvinellidae showed the opposite (Supplementary Fig. 2). Codons with an uracil at the 3rd position were used less often, while 4-fold degenerated codons ending on adenine and 2-fold ones ending on cytosine were used more often. Additionally, tRNAs of mitochondrial origin with 2-fold degenerated codons ending on guanine were used more often and, vice versa, 2-fold ones ending on adenine less often.

## *References*


Bleidorn C, Kruse I, Albrecht S, Bartolomaeus T. 2006a. Mitochondrial sequence data expose the putative cosmopolitan polychaete *Scoloplos armiger* (Annelida, Orbiniidae) as a species complex. BMC Evol. Biol., 6:47.

Bleidorn C, Podsiadlowski L, Bartolomaeus T. 2006b. The complete mitochondrial genome of the orbiniid polychaete *Orbinia latreilli* (Annelida, Orbiniidae) - a novel gene order for Annelida and implications for annelid phylogeny. Gene, 370:96-103.

Boore JL, Brown WM. 2000. Mitochondrial genomes of *Galathealinum*, *Helobdella* and *Platynereis*: Sequence and gene arrangements comparisons indicate that Pogonophora is not a phylum and Annelida and Arthropoda are not sister taxa. Mol. Biol. Evol., 17:87-106.

Boore JL, Staton JL. 2002. The Mitochondrial Genome of the Sipunculid *Phascolopsis gouldii* Supports Its Association with Annelida Rather than Mollusca. Mol. Biol. Evol., 19:127-137.

Burland TG. 2000. DNASTAR's lasergene sequence analysis software. Methods Mol. Biol., 132:71-91.

Jennings RM, Halanych KM. 2005. Mitochondial genomes of *Clymenella torquata* (Maldanidae) and *Rifta pachyptila* (Siboglinidae): Evidence for conserved gene order in Annelida. Mol. Biol. Evol., 22:210-222.

Maxmen AB, King BF, Cutler EB, Giribet G. 2003. Evolutionary relationship within the protostome phylum Sipuncula: a molecular analysis o ribosomal genes and histone H3 sequence data. Mol. Phylogenet. Evol., 22:489-503.

Mwinyi A, Meyer A, Bleidorn C, Lieb B, Bartolomaeus T, Podsiadlowski L. 2009. Mitochondrial genome sequence and gene order of *Sipunculus nudus* give additional support for an inclusion of Sipuncula into Annelida. BMC Genomics, 10:27.

Schulze A, Cutler E, Giribet G. 2005. Reconstructing the phylogeny of the Sipuncula. Hydrobiologia, 535-536:277-296.

Shen X, Ma X, Ren J, Zhao F. 2009. A close phylogenetic relationship between Sipuncula and Annelida evidenced from the complete mitochondrial genome sequence of *Phascolosoma esculenta*. BMC Genomics, 10:136.

Zhong M, Struck TH, Halanych KM. 2008. Phylogenetic information from three mitochondrial genomes of Terebelliformia (Annelida) worms and duplication of the methionine tRNA. Gene, 416:11-21.

SUPPLEMENTARY TABLE 1: Primers used in long-run PCR amplifications

| Fragments | Primer name | Sequence | Annealing Temp. |
|---|---|---|---|
| *A. crinita* | | | |
| *mLSU -cox1* | 16S-Ac-longF | 5'—GCG GTA TCC TGA CTG TGC TAA GGT AGC GTG—3' | 53 |
| | CO1-Ac-longR | 5'—AGA ATG AGC AAT ATT ACT AGA TAA GGG CGG—3' | 53 |
| *cox1-cob* | CO1-Ac-longF | 5'—TTA ATT CGT GTT GAG CTT GGA CAG CCA GGC-3' | 53 |
| | Cytb-Ac-longR | 5'—AGA GGG GTT ACT AGT GGA TTT GCT AAT AGC C—3' | 53 |
| *cob-nad5* | Cytb-Ac-longF | 5'—ATT TTC TTG TGC CTT TTA TTA TGG TAG CAC—3' | 54 |
| | Nad5-Ac-789R | 5'—ATC GCA CTC TAC CAA AGC TGA AAA CGC AGC—3' | 54 |
| *nad5-mLSU* | Nad5-Ac-645F | 5'—CGC TAG TTC ATT CGT CTA CAC TTG TAA CAG C—3' | -- |
| | 16S-Ac-longR | 5'—TGT CCC ACA CTT ACA TTC AGG TAT TTT CAC C—3' | -- |
| *P. sulfincola* | | | |
| *mLSU-cox1* | 16S-PS-longF | 5'—GTT CTA ACT GCA TAT CAA GGC AAA ACC AGC C—3' | 58 |
| | CO1-PS-longR | 5'—GCC TGC GTG TGC CAT GTT TCC TGC TAG TGG—3' | 58 |
| *cox1-cob* | CO1-PS-longF1 | 5'—AAT CTA CCC ACC ACT AGC AGG AAA CAT GGC—3' | 55 |
| | cob-PS-longR | 5'—AAT TGA TCA GTT TTC TGG TTC TCC CAG GGC—3' | 55 |
| | | 5' —TGR GGN TAT CAR CCN GAR CG—3' | |
| *cob-nad5* | cob-PS-longF | 5'—ACA ACC CTC TTG GTA TTA ACT CAG ACT CCG—3' | -- |
| | Nad5-PS-longR | 5'—AGT GTG GAT GAG TGG ACT AGT GCA GAG ACG—3' | -- |
| *nad5-mLSU* | Nad5-PS-longF | 5'—GAC TTC CAG CAG CAA TGG CAG CAC CTA CGC—3' | 58 |
| | 16S-PS-longR | 5'—ATC AGT TGT GCT TGT GTG GCT GGT TTT GCC—3' | 58 |
| *P. gouldi* | | | |
| *mLSU -cox1* | 16S-PecG-longF | 5'—AAA TCA TAG GAC AAG AAG ACC CCG TAG AGC—3' | 58 |
| | CO1-PecG-longR | 5'—AAA AAT AGC AAG ATC CAC GGA AGG GCC TGC—3' | 58 |
| *cox1-cox3* | CO1-PecG-longF | 5'—CTT AAT TCG TGT AGA ACT TGG TCA ACC AGG C—3' | 52 |
| | CO3-Ann-724R | 5'—ACR TCS ACA AAR TGT CAR TAY CA—3' | 52 |
| *cox3-cob* | CO3-PG-long627F | 5'—GAT TCC ACG GGC TTC ATG TTC TAA TTG GC—3' | 52 |
| | Cytb 876R | 5'—GCR TAW GCR AAW ARR AAR TAY CAY—3' | 52 |
| *cob-nad5* | Cob-PG-midF | 5'—TTG CTT CGA AAC CTT CAT GCT AAC GGA GC—3' | -- |
| | Nad5-PG-675R | 5'—AAT CGG ACT AAA AGA AAC ACT CCT GCC G—3' | -- |
| *nad5-nad4* | Nad5-PG-453F | 5'—TTA TAC TTC TTT TAT CTA TTG GAT GAG CCC—3' | 53 |
| | Nad4-PecG-longR | 5'—ATT GGG AGG AGT TAA AAG AGT AAA GGA TTG C—3' | 53 |
| *nad4-mLSU* | Nad4-PecG-longF | 5'—TTT AAC TCC TCC CAA TCC CTA TCT TTT AGC—3' | 58 |
| | 16S-PecG-longR | 5'—GTT TAG GTT AGG CGG GAT GCC TTA TTG CTC—3' | 58 |

SUPPLEMENTARY TABLE 2: Primers used for sequencing the mitochondrial genomes of *Auchenoplax crinita, Paravinella sulfincola* and *Pectinaria gouldi*.

| **Fragments** | **Primer name** | **Sequence** |
|---|---|---|
| *A. crinita* | | |
| *mLSU -cox1* | CO1-Auc1-startR | 5'—GTC GCA TAT AAC CAA CGC—3' |
| | 16S-Auc1-560F | 5'—AAT CCT ACA TGA GCT GAG—3' |
| | 16S-Auc1-midF | 5'—AAT CCT ACA TGA GCT GAG—3' |
| | Nad2-Auc1-705R | 5'—CTA CTT GTC TAG ATC TTC C—3' |
| | Nd2-Auc-189R | 5'—CAA CTC AAA TAG CTA GCC—3' |
| | Nd2-Auc-508R | 5'—TAA TAC TAC TTG TCT AGA TC—3' |
| | Nad1-Auc-37F | 5'—TTT ATG CGC TAT ACT AGC—3' |
| | Nad3-Auc1-startR | 5'—AGA AAT AAC CAT TAA CGC C—3' |
| | Nad1-Auc1-561F | 5'—TGT TTC GGT TTT AGC TGA G—3' |
| | Nad1-Auc1-521R | 5'—GAT AAT CAA ACT TGC CG—3' |
| | Nad2-Auc-599R | 5'—AAT TAT TGA CCC TGC TAC—3' |
| | Nad2-Auc-588F | 5'—AAT TTC TAG TGT TAG AGC—3' |
| | Nad1-Auc-startF | 5'—TGG CAG ACT AGT GCG TTG G—3' |
| | Nad2-A1-5R | 5'—AGG TTT TCC ATG TTA ATG C—3' |
| | Nd1-A1-500F | 5'—TGT TTC GGT TTT AGC TGA G—3' |
| *cox1-cob* | CO1-Auc1-591F | 5'—TTC GTT ACC AGT ACT GGC—3' |
| | Cytb-Auc1-417R | 5'—CCA TAA TAA ATT CCA CGC—3' |
| | Cob-Auc1-mid2R | 5'—ACA TAC ACG CTG AGT AAC—3' |
| | CO3-Auc1-middR | 5'—CTA CTG GCG GTC ATA CAC—3' |
| | Nad6-Auc1-156R | 5'—TTC ATG ACG TTG ATT AGG—3' |
| | CO1-Auc1-1004F | 5'—AAA ATA GTT TAT GAT CCT GC—3' |
| | Atp8-Auc1-startR1 | 5'—AAT AGG AGA CAA ATG AGG C—3' |
| | CO1-Auc-911R | 5'—TTG ACA AAA TAA TCC CAG—3' |
| | Nad6-Auc-startR | 5'—AAT CAA AAC CCT ATG TGC—3' |
| | Nad6-Auc-45F | 5'—TAG TTT TAG TAT TAC TTG G—3' |
| | Atp8-A1-endR | 5'—TAA TAG GAG ACA AAT GAG GC—3' |
| | CO3-A1-startR | 5'—TAC TGT CAC ACC AGA AGC—3' |
| *cob-nad5* | Cob-Auc1-942F | 5'—AGC AGA AGC GTA GAA GAC —3' |
| | Nd5-Auc1-436R | 5'—AAC TAT GAT TAT CCC AGC—3' |
| | Atp6-Auc1-70F | 5'—AGT TTC TGT AAT AGG AGT G—3' |
| | Cob-Auc1-enddR | 5'—AAA GTA CAC ATA AAG CTC C—3' |
| | Nad4-Auc-812R | 5'—ACA AAC TTC CAT GTT GAG—3' |
| | Nad5-Auc-middR | 5'—GAA GTT ATT ATC CCA GGC—3' |
| | Nad5-Auc-middF | 5'—TTA ACT TAT ATG GCG TAT G—3' |
| | Nad4-Auc1-672R | 5'—TAG ACC CAG CAA CAG GAG C—3' |
| | tRNAx-Auc-befNd5R | 5'—AAC ATC GAA ACC ATG GGC—3' |
| | Atp6-Auc-220F | 5'—TAT GTT TAC TCA GTC ACG—3' |
| | Atp6-Auc-235R | 5'—ACG CTG ATA AAA CAC TTC—3' |
| | Nad4-A1-312R | 5'—CTA AAA TTA TAG ACC CAG C—3' |
| | Atp6-A1-530F | 5'—TAA GAG CAG GGC ATA TTG CG—3' |
| *P. sulfincola* | | |
| *mLSU-cox1* | 16S-PS-midF | 5'—CTA CCT GCT ACA GTT CTC C —3' |
| | 16S-PS-endF | 5'—AAA ATA TGC CCT ACT AGG CTC—3' |
| | tRNAX-PS-R | 5'—TTA TGA AGA CGA CTT TGA GG—3' |
| | Nad2-PS-235R | 5'—GGA AAC CAT TGA TGA CAC GG—3' |

| | | |
|---|---|---|
| | Nad1-PS-endF | 5'—TGA ACG GAT AGC TCT GAT GC—3' |
| | Nad3-PS-211R | 5'—TGT GGC TGA GTG CTT AGA CG—3' |
| | Nad1-PS-308R | 5'—GTT GAT ATT GGA TAA TTG GC—3' |
| *cox1-cob* | CO1-PS-834F | 5'—AGA CAC ACG AGC CTA CTT TAC CGC —3' |
| | Cob-PS-202R | 5'—TGA GAA GGC TAG GTC TAC ATT TGG—3' |
| | CO1-PS-1500F | 5'—CAC CAG CAT TCC ACT CAG GAG CCG—3' |
| | Nad6-PS-108R | 5'—GTG TTA GAG TCG ATA GTC TGC—3' |
| | CO2-PS-497F | 5'—AGC AGA CGT AAT TCA TTC ATG AGC C—3' |
| | CO3-PS-333R | 5'—TTA AGA GTG GTA CTG CAA ATG GGT C—3' |
| *nad5-mLSU* | 12S-PS-endR | 5'—ATG CAG TGA CAT GGT GGC TTG CTG CGG —3' |
| | Nad5-PS-1160F | 5'—GTG CCG CAG CTG CCT ACT CAA CAC G —3' |
| | 12S-PS-startR | 5'—TTC AGT GTA AGT GAG TGG CAT CAC C—3' |
| | tRNAX-PS-R2 | 5'—TCT ATT TGG ACA TTT CGT TAA ACC G—3' |
| | Nad4-PS-200F | 5'—ATT CAT CAT TGT AGT CTC TTT CGC—3' |
| | Nad4-PS-531F | 5'—CGT TGC CGC ATC TTT ACC GC—3' |
| | Nad4-PS-1176R | 5'—ATA AGA TTA AAG AGT ATG TAC C—3' |
| *P. gouldi* | | |
| *mLSU -cox1* | 16S-PG-720F | 5'—GCC CAG CTA ATT GGC AGA CTA GTG C —3' |
| | CO1-PG-startR | 5'—GAG ATA ACG TGG CAC AAA CCA AAG C—3' |
| | Nad1-PG-261F | 5'—TCT GCG CTT TAA CCT TAG CCC TGC—3' |
| | Nad2-PG-495R | 5'—CCC GTA TTT GGG TTT GAT TTA GC—3' |
| | Nad1-PG-656F | 5'—ATT GAA TAT AGA AGT GGC AGC—3' |
| | Nad2-PG-2R | 5'—TTT ATA GGG GCA GTC ATA GG—3' |
| | Nad2-PG-321F | 5'—GTA CAA TTT CAA TCA ACC AG—3' |
| *cox1-cox3* | CO1-PG-900R | 5'—CAT GGT TGC GGC AGT AAA ATA GGC —3' |
| | CO3-PG-204R | 5'—CCT AAA AAA GTT CCC TCA CG —3' |
| | CO2-PG-591R | 5'—GCA TTG ACC ATA GAA TAC TCC G—3' |
| | CO2-PG-startR | 5'—TCT TGG AAG GAT AGT TGG GCT C—3' |
| | CO1-PG-1257F | 5'—TTC TTC CCT CAA CAC TTC CTC GG—3' |
| | CO1-PG-1134R | 5'—AGA GGA AAC CAG TGG TTA AAT GC—3' |
| *cox3-cob* | Cob-PG-midF | 5'—TTG CTT CGA AAC CTT CAT GCT AAC GGA GC —3' |
| | Nad6-PG-273F | 5'—CTA ATT GCA CTC CTT TTG TCA GGC—3' |
| | CO3-PG-L627F | 5'—GAT TCC ACG GGC TTC ATG TTC TAA TTG GC —3' |
| *nad5-nad4* | Nad4-PG-186F | 5'—TCT TAA CCT TAT GAA TTT CCA GCC—3' |
| | Nad4-PG-516R | 5'—TTT ATG AGG ATC GAG TAA CTT GC—3' |
| | Nad4L-PG-75F | 5'—ATG GCG ACC CTG ATT ATT CAA CG—3' |
| | Nd5-PG-1005R | 5'—GTT TGC AAT TAA AAG GCA GGA C—3' |
| | Nd5-PG-1065F | 5'—GCC GGG TTC TAC TCT AAA GAC C—3' |
| | Nd5-PG-1587F | 5'—CAA GGT TGA AAC GAA ATA GCC GG—3' |
| | Nad4-PG-453R | 5'—CTA TAA GGA GTG GTA GTG AAG C—3' |
| *nad4-mLSU* | Nad4-PG-846F | 5'—CTT ATT GCT TAT TCC TCA GTT GGT C —3' |
| | Nad4-PG-1026F | 5'—GGG TTA CTC ATT AAT ACT TTC CAG—3' |
| | 12S-PG-startR | 5'—AAT TTT TCA ATA GTT TAC TCA TGG AG —3' |
| | tRNAX-PG-F | 5'—CAT GAC CCA AAA GTG GAA ACA ATC C—3' |
| | 12S-PG-111F | 5'—TGT CCT CTA ACT CGA TAA TCC ACG—3' |
| | 12S-PG-midR | 5'—ATT CCT GAT GCT ATG CTT TGT GGC—3' |
| | Nad4-PG-519R | 5'—TGG CTG GAA AGT ATA TGA GTA ACC—3' |

SUPPLEMENTARY TABLE 3: RSCU analyses for the three Terebelliformia taxa for which we obtained new mitochondrial data.

| **AA** | **Codon** | ***A. crinita*** | | ***P. gouldi*** | | ***P. sulfincola*** | | **AA** | **Codon** | ***A. crinita*** | | ***P. gouldi*** | | ***P. sulfincola*** | |
|---|---|---|---|---|---|---|---|---|---|---|---|---|---|---|---|
| Ala | GCA | 63 | 1.223 | 63 | 0.966 | 141 | 2.029 | Lys | AAA | 45 | 1.286 | 61 | 1.584 | 62 | 1.797 |
| | GCC | 2 | 0.039 | 94 | 1.441 | 82 | 1.180 | | AAG | 25 | 0.714 | 16 | 0.416 | 7 | 0.203 |
| | GCG | 41 | 0.796 | 15 | 0.230 | 6 | 0.086 | Met | AUA | 165 | 1.480 | 147 | 1.531 | 105 | 1.160 |
| | GCU | 100 | 1.942 | 89 | 1.364 | 49 | 0.705 | | AUG | 58 | 0.520 | 45 | 0.469 | 76 | 0.840 |
| Arg | CGA | 21 | 1.200 | 36 | 2.361 | 29 | 2.035 | Phe | UUC | 13 | 0.089 | 89 | 0.695 | 126 | 1.135 |
| | CGC | 1 | 0.057 | 5 | 0.328 | 12 | 0.842 | | UUU | 280 | 1.911 | 167 | 1.305 | 96 | 0.865 |
| | CGG | 15 | 0.857 | 6 | 0.393 | 2 | 0.140 | Pro | CCA | 44 | 1.313 | 49 | 1.153 | 104 | 2.337 |
| | CGU | 33 | 1.886 | 14 | 0.918 | 14 | 0.982 | | CCC | 2 | 0.060 | 55 | 1.294 | 19 | 0.427 |
| Asn | AAC | 11 | 0.227 | 60 | 1.091 | 83 | 1.495 | | CCG | 8 | 0.239 | 15 | 0.353 | 20 | 0.449 |
| | AAU | 86 | 1.773 | 50 | 0.909 | 28 | 0.505 | | CCU | 80 | 2.388 | 51 | 1.200 | 35 | 0.787 |
| Asp | GAC | 14 | 0.424 | 31 | 1.016 | 31 | 1.000 | Ser | AGA | 88 | 2.071 | 38 | 0.897 | 39 | 1.091 |
| | GAU | 52 | 1.576 | 30 | 0.984 | 31 | 1.000 | | AGC | 5 | 0.118 | 13 | 0.307 | 17 | 0.476 |
| Cys | UGC | 11 | 0.415 | 15 | 1.000 | 21 | 1.135 | | AGG | 38 | 0.894 | 7 | 0.165 | 8 | 0.224 |
| | UGU | 42 | 1.585 | 15 | 1.000 | 16 | 0.865 | | AGU | 38 | 0.894 | 18 | 0.425 | 12 | 0.336 |
| Gln | CAA | 33 | 1.294 | 56 | 1.723 | 61 | 1.694 | | UCA | 62 | 1.459 | 68 | 1.605 | 90 | 2.517 |
| | CAG | 18 | 0.706 | 9 | 0.277 | 11 | 0.306 | | UCC | 6 | 0.141 | 68 | 1.605 | 41 | 1.147 |
| Glu | GAA | 39 | 1.000 | 50 | 1.493 | 45 | 1.429 | | UCG | 16 | 0.376 | 10 | 0.236 | 14 | 0.392 |
| | GAG | 39 | 1.000 | 17 | 0.507 | 18 | 0.571 | | UCU | 87 | 2.047 | 117 | 2.761 | 65 | 1.818 |
| Gly | GGA | 52 | 0.908 | 54 | 1.207 | 78 | 1.880 | Ter(.) | UAA/U | 12 | 2.000 | 9 | 1.800 | 9 | 1.636 |
| | GGC | 12 | 0.210 | 41 | 0.916 | 38 | 0.916 | | UAG | 0 | 0.000 | 1 | 0.200 | 2 | 0.364 |
| | GGG | 121 | 2.114 | 43 | 0.961 | 19 | 0.458 | Thr | ACA | 63 | 1.839 | 59 | 1.198 | 150 | 2.317 |
| | GGU | 44 | 0.769 | 41 | 0.916 | 31 | 0.747 | | ACC | 4 | 0.117 | 65 | 1.320 | 58 | 0.896 |
| His | CAC | 7 | 0.203 | 51 | 1.133 | 67 | 1.489 | | ACG | 8 | 0.234 | 4 | 0.081 | 12 | 0.185 |
| | CAU | 62 | 1.797 | 39 | 0.867 | 23 | 0.511 | | ACU | 62 | 1.810 | 69 | 1.401 | 39 | 0.602 |
| Ile | AUC | 21 | 0.166 | 73 | 0.638 | 105 | 0.847 | Trp | UGA | 53 | 1.082 | 66 | 1.451 | 68 | 1.563 |
| | AUU | 232 | 1.834 | 156 | 1.362 | 143 | 1.153 | | UGG | 45 | 0.918 | 25 | 0.549 | 19 | 0.437 |
| Leu | CUA | 32 | 0.396 | 111 | 1.271 | 194 | 2.456 | Tyr | UAC | 15 | 0.219 | 45 | 0.891 | 59 | 1.311 |
| | CUC | 2 | 0.025 | 71 | 0.813 | 58 | 0.734 | | UAU | 122 | 1.781 | 56 | 1.109 | 31 | 0.689 |
| | CUG | 10 | 0.124 | 24 | 0.275 | 54 | 0.684 | Val | GUA | 117 | 1.505 | 63 | 1.527 | 91 | 1.906 |
| | CUU | 57 | 0.705 | 153 | 1.752 | 88 | 1.114 | | GUC | 7 | 0.090 | 36 | 0.873 | 28 | 0.586 |
| | UUA | 321 | 3.971 | 137 | 1.569 | 67 | 0.848 | | GUG | 48 | 0.617 | 17 | 0.412 | 15 | 0.314 |
| | UUG | 63 | 0.779 | 28 | 0.321 | 13 | 0.165 | | GUU | 139 | 1.788 | 49 | 1.188 | 57 | 1.194 |
| 2fold | NNA | 57.8 | 1.357 | 64.8 | 1.597 | 58.3 | 1.547 | 4fold | NNA | 60.0 | 1.332 | 54.0 | 1.402 | 98.8 | 2.084 |
| | NNG | 30.8 | 0.643 | 18.8 | 0.403 | 22.2 | 0.453 | | NNC | 4.7 | 0.095 | 49.3 | 1.029 | 39.5 | 0.808 |
| | NNC | 13.1 | 0.249 | 52.0 | 0.924 | 70.3 | 1.202 | | NNG | 40.2 | 0.809 | 16.7 | 0.405 | 12.3 | 0.272 |
| | NNU | 125.1 | 1.751 | 73.3 | 1.076 | 52.6 | 0.798 | | NNU | 76.3 | 1.764 | 52.2 | 1.165 | 37.5 | 0.836 |

SUPPLEMENTARY TABLE 4: RSCU analyses for the other taxa in our analyses.

| AA | Codon | *Nephtys* sp. | | *G. brachiosum* | | *H. robusta* | | *C. torquata* | | *S.* cf. *armiger* | | *O. latreillii* | | *U. caupo* | | *L. terrestris* | | *R. pachyptila* | | *Phascolopsis gouldi* | | *P. dumerilii* | |
|---|---|---|---|---|---|---|---|---|---|---|---|---|---|---|---|---|---|---|---|---|---|---|---|
| Ala | GCA | 69 | 0.914 | 36 | 1.674 | 58 | 1.771 | 100 | 1.581 | 90 | 1.545 | 78 | 1.139 | 121 | 1.698 | 81 | 1.178 | 68 | 1.439 | 48 | 1.371 | 90 | 1.343 |
| | GCC | 101 | 1.338 | 7 | 0.326 | 31 | 0.947 | 58 | 0.917 | 71 | 1.219 | 112 | 1.635 | 75 | 1.053 | 80 | 1.164 | 51 | 1.079 | 32 | 0.914 | 68 | 1.015 |
| | GCG | 17 | 0.225 | 0 | 0.000 | 5 | 0.153 | 6 | 0.095 | 3 | 0.052 | 6 | 0.088 | 14 | 0.196 | 15 | 0.218 | 1 | 0.021 | 5 | 0.143 | 30 | 0.448 |
| | GCU | 115 | 1.523 | 43 | 2.000 | 37 | 1.130 | 89 | 1.407 | 69 | 1.185 | 78 | 1.139 | 75 | 1.053 | 99 | 1.440 | 69 | 1.460 | 55 | 1.571 | 80 | 1.194 |
| Arg | CGA | 22 | 1.397 | 14 | 1.436 | 29 | 2.900 | 41 | 2.645 | 40 | 2.712 | 33 | 2.063 | 46 | 2.788 | 32 | 2.032 | 38 | 2.868 | 22 | 2.095 | 25 | 1.724 |
| | CGC | 17 | 1.079 | 3 | 0.308 | 5 | 0.500 | 4 | 0.258 | 8 | 0.542 | 8 | 0.500 | 5 | 0.303 | 7 | 0.444 | 5 | 0.377 | 5 | 0.476 | 13 | 0.897 |
| | CGG | 6 | 0.381 | 1 | 0.103 | 0 | 0.000 | 4 | 0.258 | 2 | 0.136 | 4 | 0.250 | 9 | 0.545 | 9 | 0.571 | 0 | 0.000 | 0 | 0.000 | 10 | 0.690 |
| | CGU | 18 | 1.143 | 21 | 2.154 | 6 | 0.600 | 13 | 0.839 | 9 | 0.610 | 19 | 1.188 | 6 | 0.364 | 15 | 0.952 | 10 | 0.755 | 15 | 1.429 | 10 | 0.690 |
| Asn | AAC | 65 | 1.008 | 12 | 0.238 | 33 | 0.667 | 70 | 0.909 | 61 | 0.961 | 59 | 0.937 | 65 | 1.040 | 66 | 0.971 | 35 | 0.654 | 27 | 0.720 | 66 | 0.957 |
| | AAU | 64 | 0.992 | 89 | 1.762 | 66 | 1.333 | 84 | 1.091 | 66 | 1.039 | 67 | 1.063 | 60 | 0.960 | 70 | 1.029 | 72 | 1.346 | 48 | 1.280 | 72 | 1.043 |
| Asp | GAC | 43 | 1.246 | 5 | 0.263 | 28 | 1.167 | 32 | 0.985 | 35 | 1.094 | 38 | 1.152 | 37 | 1.254 | 34 | 0.919 | 25 | 0.943 | 18 | 0.857 | 37 | 1.121 |
| | GAU | 26 | 0.754 | 33 | 1.737 | 20 | 0.833 | 33 | 1.015 | 29 | 0.906 | 28 | 0.848 | 22 | 0.746 | 40 | 1.081 | 28 | 1.057 | 24 | 1.143 | 29 | 0.879 |
| Cys | UGC | 9 | 0.600 | 4 | 0.364 | 6 | 0.600 | 16 | 1.000 | 22 | 1.128 | 24 | 1.067 | 11 | 0.759 | 21 | 1.077 | 13 | 0.839 | 7 | 0.778 | 13 | 0.619 |
| | UGU | 21 | 1.400 | 18 | 1.636 | 14 | 1.400 | 16 | 1.000 | 17 | 0.872 | 21 | 0.933 | 18 | 1.241 | 18 | 0.923 | 18 | 1.161 | 11 | 1.222 | 29 | 1.381 |
| Gln | CAA | 54 | 1.543 | 37 | 2.000 | 42 | 1.953 | 74 | 1.873 | 74 | 1.873 | 75 | 1.829 | 84 | 1.750 | 55 | 1.719 | 55 | 1.897 | 40 | 1.905 | 58 | 1.589 |
| | CAG | 16 | 0.457 | 0 | 0.000 | 1 | 0.047 | 5 | 0.127 | 5 | 0.127 | 7 | 0.171 | 12 | 0.250 | 9 | 0.281 | 3 | 0.103 | 2 | 0.095 | 15 | 0.411 |
| Glu | GAA | 55 | 1.507 | 48 | 2.000 | 39 | 1.592 | 60 | 1.600 | 57 | 1.932 | 64 | 1.882 | 73 | 1.759 | 41 | 1.155 | 58 | 1.871 | 31 | 1.771 | 60 | 1.481 |
| | GAG | 18 | 0.493 | 0 | 0.000 | 10 | 0.408 | 15 | 0.400 | 2 | 0.068 | 4 | 0.118 | 10 | 0.241 | 30 | 0.845 | 4 | 0.129 | 4 | 0.229 | 21 | 0.519 |
| Gly | GGA | 74 | 1.495 | 69 | 2.464 | 47 | 1.679 | 69 | 1.401 | 96 | 2.313 | 82 | 1.763 | 126 | 2.483 | 51 | 1.103 | 101 | 2.525 | 47 | 1.694 | 77 | 1.548 |
| | GGC | 66 | 1.333 | 3 | 0.107 | 12 | 0.429 | 39 | 0.792 | 25 | 0.602 | 62 | 1.333 | 27 | 0.532 | 34 | 0.735 | 19 | 0.475 | 24 | 0.865 | 48 | 0.965 |
| | GGG | 36 | 0.727 | 1 | 0.036 | 23 | 0.821 | 55 | 1.117 | 28 | 0.675 | 12 | 0.258 | 36 | 0.709 | 62 | 1.341 | 10 | 0.250 | 15 | 0.541 | 48 | 0.965 |
| | GGU | 22 | 0.444 | 39 | 1.393 | 30 | 1.071 | 34 | 0.690 | 17 | 0.410 | 30 | 0.645 | 14 | 0.276 | 38 | 0.822 | 30 | 0.750 | 25 | 0.901 | 26 | 0.523 |
| His | CAC | 48 | 0.950 | 5 | 0.175 | 24 | 0.842 | 30 | 0.714 | 40 | 0.930 | 60 | 1.111 | 55 | 1.250 | 59 | 1.242 | 22 | 0.620 | 32 | 0.914 | 50 | 1.111 |
| | CAU | 53 | 1.050 | 52 | 1.825 | 33 | 1.158 | 54 | 1.286 | 46 | 1.070 | 48 | 0.889 | 33 | 0.750 | 36 | 0.758 | 49 | 1.380 | 38 | 1.086 | 40 | 0.889 |
| Ile | AUC | 58 | 0.404 | 33 | 0.254 | 64 | 0.601 | 91 | 0.543 | 85 | 0.526 | 123 | 0.769 | 100 | 0.667 | 105 | 0.702 | 59 | 0.463 | 44 | 0.611 | 83 | 0.502 |
| | AUU | 229 | 1.596 | 227 | 1.746 | 149 | 1.399 | 244 | 1.457 | 238 | 1.474 | 197 | 1.231 | 200 | 1.333 | 194 | 1.298 | 196 | 1.537 | 100 | 1.389 | 248 | 1.498 |
| Leu | CUA | 81 | 0.822 | 39 | 0.711 | 99 | 1.880 | 151 | 1.601 | 178 | 1.884 | 223 | 2.172 | 223 | 2.295 | 177 | 1.907 | 127 | 1.558 | 56 | 1.249 | 136 | 1.603 |
| | CUC | 90 | 0.914 | 6 | 0.109 | 22 | 0.418 | 54 | 0.572 | 88 | 0.931 | 117 | 1.140 | 62 | 0.638 | 74 | 0.797 | 45 | 0.552 | 40 | 0.892 | 31 | 0.365 |
| | CUG | 17 | 0.173 | 1 | 0.018 | 7 | 0.133 | 14 | 0.148 | 8 | 0.085 | 15 | 0.146 | 38 | 0.391 | 32 | 0.345 | 5 | 0.061 | 2 | 0.045 | 19 | 0.224 |
| | CUU | 201 | 2.041 | 77 | 1.404 | 48 | 0.911 | 109 | 1.155 | 146 | 1.545 | 161 | 1.568 | 105 | 1.081 | 95 | 1.023 | 116 | 1.423 | 80 | 1.784 | 100 | 1.179 |
| | UUA | 188 | 1.909 | 203 | 3.702 | 128 | 2.430 | 226 | 2.396 | 141 | 1.492 | 98 | 0.955 | 145 | 1.492 | 150 | 1.616 | 191 | 2.344 | 91 | 2.030 | 191 | 2.251 |
| | UUG | 14 | 0.142 | 3 | 0.055 | 12 | 0.228 | 12 | 0.127 | 6 | 0.063 | 2 | 0.019 | 10 | 0.103 | 29 | 0.312 | 5 | 0.061 | 0 | 0.000 | 32 | 0.377 |
| 2fold | NNA | 73.167 | 1.587 | 58.000 | 1.917 | 54.000 | 1.740 | 90.333 | 1.768 | 83.167 | 1.832 | 84.500 | 1.821 | 81.500 | 1.614 | 75.000 | 1.571 | 67.667 | 1.822 | 38.833 | 1.890 | 84.333 | 1.649 |
| | NNG | 18.833 | 0.413 | 2.500 | 0.083 | 9.500 | 0.260 | 12.167 | 0.232 | 5.667 | 0.168 | 9.333 | 0.179 | 18.833 | 0.386 | 24.833 | 0.429 | 6.500 | 0.178 | 2.500 | 0.110 | 21.667 | 0.351 |
| | NNC | 49.714 | 0.800 | 14.286 | 0.249 | 35.000 | 0.760 | 51.000 | 0.766 | 56.143 | 0.920 | 72.143 | 1.045 | 63.857 | 1.003 | 106.714 | 1.053 | 38.571 | 0.690 | 28.286 | 0.750 | 53.714 | 0.826 |
| | NNU | 95.429 | 1.200 | 102.286 | 1.751 | 64.857 | 1.240 | 104.571 | 1.234 | 83.143 | 1.080 | 74.143 | 0.955 | 76.429 | 0.997 | 82.286 | 0.947 | 81.571 | 1.310 | 51.714 | 1.250 | 92.286 | 1.174 |

| AA | Codon | *Nephtys* sp. | | *G. brachiosum* | | *H. robusta* | | *C. torquata* | | *S.* cf. *armiger* | | *O. latreillii* | | *U. caupo* | | *L. terrestris* | | *R. pachyptila* | | *Phascolopsis gouldi* | | *P. dumerilii* | |
|---|---|---|---|---|---|---|---|---|---|---|---|---|---|---|---|---|---|---|---|---|---|---|---|
| Lys | AAA | 64 | 1.662 | 56 | 1.898 | 48 | 1.714 | 82 | 1.907 | 90 | 1.957 | 84 | 1.931 | 77 | 1.692 | 70 | 1.522 | 62 | 1.908 | 32 | 1.882 | 66 | 1.692 |
| | AAG | 13 | 0.338 | 3 | 0.102 | 8 | 0.286 | 4 | 0.093 | 2 | 0.043 | 3 | 0.069 | 14 | 0.308 | 22 | 0.478 | 3 | 0.092 | 2 | 0.118 | 12 | 0.308 |
| Met | AUA | 156 | 1.529 | 137 | 1.851 | 129 | 1.613 | 233 | 1.792 | 178 | 1.816 | 174 | 1.649 | 170 | 1.532 | 194 | 1.504 | 138 | 1.683 | 66 | 1.970 | 233 | 1.596 |
| | AUG | 48 | 0.471 | 11 | 0.149 | 31 | 0.388 | 27 | 0.208 | 18 | 0.184 | 37 | 0.351 | 52 | 0.468 | 64 | 0.496 | 26 | 0.317 | 1 | 0.030 | 59 | 0.404 |
| Phe | UUC | 78 | 0.538 | 33 | 0.262 | 50 | 0.595 | 74 | 0.503 | 103 | 0.880 | 134 | 1.085 | 115 | 0.833 | 400 | 1.429 | 93 | 0.791 | 46 | 0.630 | 82 | 0.678 |
| | UUU | 212 | 1.462 | 219 | 1.738 | 118 | 1.405 | 220 | 1.497 | 131 | 1.120 | 113 | 0.915 | 161 | 1.167 | 160 | 0.571 | 142 | 1.209 | 100 | 1.370 | 160 | 1.322 |
| Pro | CCA | 20 | 0.455 | 37 | 1.437 | 75 | 2.970 | 80 | 1.730 | 66 | 1.412 | 74 | 1.430 | 80 | 1.584 | 53 | 1.184 | 46 | 1.150 | 40 | 1.553 | 64 | 1.497 |
| | CCC | 57 | 1.295 | 5 | 0.194 | 4 | 0.158 | 18 | 0.389 | 43 | 0.920 | 51 | 0.986 | 44 | 0.871 | 55 | 1.229 | 34 | 0.850 | 20 | 0.777 | 52 | 1.216 |
| | CCG | 3 | 0.068 | 1 | 0.039 | 2 | 0.079 | 13 | 0.281 | 6 | 0.128 | 5 | 0.097 | 6 | 0.119 | 16 | 0.358 | 1 | 0.025 | 2 | 0.078 | 8 | 0.187 |
| | CCU | 96 | 2.182 | 60 | 2.330 | 20 | 0.792 | 74 | 1.600 | 72 | 1.540 | 77 | 1.488 | 72 | 1.426 | 55 | 1.229 | 79 | 1.975 | 41 | 1.592 | 47 | 1.099 |
| Ser | AGA | 39 | 0.886 | 39 | 1.357 | 25 | 1.047 | 65 | 1.600 | 46 | 1.067 | 44 | 1.020 | 62 | 1.455 | 63 | 1.381 | 53 | 1.477 | 23 | 1.278 | 57 | 1.378 |
| | AGC | 24 | 0.545 | 1 | 0.035 | 5 | 0.209 | 12 | 0.295 | 3 | 0.070 | 14 | 0.325 | 15 | 0.352 | 14 | 0.307 | 6 | 0.167 | 5 | 0.278 | 26 | 0.628 |
| | AGG | 8 | 0.182 | 3 | 0.104 | 5 | 0.209 | 13 | 0.320 | 4 | 0.093 | 2 | 0.046 | 7 | 0.164 | 13 | 0.285 | 3 | 0.084 | 0 | 0.000 | 19 | 0.459 |
| | AGU | 15 | 0.341 | 6 | 0.209 | 11 | 0.461 | 16 | 0.394 | 9 | 0.209 | 17 | 0.394 | 7 | 0.164 | 20 | 0.438 | 6 | 0.167 | 4 | 0.222 | 25 | 0.604 |
| | UCA | 59 | 1.341 | 57 | 1.983 | 83 | 3.476 | 107 | 2.634 | 130 | 3.014 | 91 | 2.110 | 74 | 1.736 | 94 | 2.060 | 83 | 2.314 | 23 | 1.278 | 78 | 1.885 |
| | UCC | 67 | 1.523 | 8 | 0.278 | 19 | 0.796 | 38 | 0.935 | 68 | 1.577 | 74 | 1.716 | 65 | 1.525 | 74 | 1.622 | 51 | 1.422 | 30 | 1.667 | 42 | 1.015 |
| | UCG | 9 | 0.205 | 1 | 0.035 | 5 | 0.209 | 3 | 0.074 | 5 | 0.116 | 8 | 0.186 | 3 | 0.070 | 8 | 0.175 | 3 | 0.084 | 0 | 0.000 | 18 | 0.435 |
| | UCU | 131 | 2.977 | 115 | 4.000 | 38 | 1.592 | 71 | 1.748 | 80 | 1.855 | 95 | 2.203 | 108 | 2.534 | 79 | 1.732 | 82 | 2.286 | 59 | 3.278 | 66 | 1.595 |
| Ter(.) | UAA/U | 10 | 1.538 | 7 | 1.750 | 7 | 1.750 | 12 | 1.846 | 9 | 1.500 | 11 | 1.692 | 9 | 1.385 | 13 | 2.000 | 8 | 1.600 | 7 | 2.000 | 13 | 2.000 |
| | UAG | 3 | 0.462 | 1 | 0.250 | 1 | 0.250 | 1 | 0.154 | 3 | 0.500 | 2 | 0.308 | 4 | 0.615 | 0 | 0.000 | 2 | 0.400 | 0 | 0.000 | 0 | 0.000 |
| Thr | ACA | 79 | 1.179 | 34 | 1.432 | 77 | 2.216 | 139 | 2.180 | 102 | 1.729 | 87 | 1.338 | 93 | 1.404 | 90 | 1.434 | 64 | 1.399 | 42 | 1.631 | 98 | 1.468 |
| | ACC | 94 | 1.403 | 6 | 0.253 | 25 | 0.719 | 44 | 0.690 | 67 | 1.136 | 90 | 1.385 | 91 | 1.374 | 70 | 1.116 | 42 | 0.918 | 23 | 0.893 | 75 | 1.124 |
| | ACG | 7 | 0.104 | 0 | 0.000 | 0 | 0.000 | 5 | 0.078 | 4 | 0.068 | 5 | 0.077 | 5 | 0.075 | 11 | 0.175 | 2 | 0.044 | 1 | 0.039 | 23 | 0.345 |
| | ACU | 88 | 1.313 | 55 | 2.316 | 37 | 1.065 | 67 | 1.051 | 63 | 1.068 | 78 | 1.200 | 76 | 1.147 | 80 | 1.275 | 75 | 1.639 | 37 | 1.437 | 71 | 1.064 |
| Trp | UGA | 100 | 1.739 | 63 | 2.000 | 59 | 1.815 | 81 | 1.588 | 91 | 1.916 | 99 | 1.941 | 76 | 1.567 | 77 | 1.525 | 85 | 1.977 | 57 | 1.810 | 76 | 1.535 |
| | UGG | 15 | 0.261 | 0 | 0.000 | 6 | 0.185 | 21 | 0.412 | 4 | 0.084 | 3 | 0.059 | 21 | 0.433 | 24 | 0.475 | 1 | 0.023 | 6 | 0.190 | 23 | 0.465 |
| Tyr | UAC | 47 | 0.855 | 8 | 0.186 | 40 | 0.851 | 44 | 0.704 | 47 | 0.922 | 67 | 1.196 | 64 | 1.219 | 62 | 1.033 | 23 | 0.517 | 24 | 0.738 | 45 | 0.796 |
| | UAU | 63 | 1.145 | 78 | 1.814 | 54 | 1.149 | 81 | 1.296 | 55 | 1.078 | 45 | 0.804 | 41 | 0.781 | 58 | 0.967 | 66 | 1.483 | 41 | 1.262 | 68 | 1.204 |
| Val | GUA | 52 | 1.112 | 39 | 2.229 | 81 | 2.551 | 86 | 2.060 | 68 | 2.211 | 80 | 2.013 | 99 | 2.041 | 27 | 0.688 | 76 | 2.321 | 42 | 1.732 | 99 | 1.895 |
| | GUC | 28 | 0.599 | 3 | 0.171 | 11 | 0.346 | 23 | 0.551 | 22 | 0.715 | 31 | 0.780 | 28 | 0.577 | 44 | 1.121 | 19 | 0.580 | 20 | 0.825 | 18 | 0.344 |
| | GUG | 10 | 0.214 | 1 | 0.057 | 6 | 0.189 | 10 | 0.240 | 4 | 0.130 | 5 | 0.126 | 18 | 0.371 | 38 | 0.968 | 2 | 0.061 | 4 | 0.165 | 23 | 0.440 |
| | GUU | 97 | 2.075 | 27 | 1.543 | 29 | 0.913 | 48 | 1.150 | 29 | 0.943 | 43 | 1.082 | 49 | 1.010 | 48 | 1.223 | 34 | 1.038 | 31 | 1.278 | 69 | 1.321 |
| 4fold | NNA | 52.667 | 1.092 | 38.167 | 1.779 | 61.167 | 2.348 | 85.833 | 1.933 | 77.000 | 1.987 | 72.333 | 1.624 | 94.167 | 2.000 | 55.667 | 1.270 | 65.500 | 1.950 | 40.167 | 1.679 | 75.500 | 1.579 |
| | NNC | 60.500 | 1.175 | 4.500 | 0.226 | 14.667 | 0.517 | 31.000 | 0.600 | 39.333 | 0.856 | 59.000 | 1.103 | 45.000 | 0.785 | 48.333 | 0.968 | 28.333 | 0.713 | 20.667 | 0.792 | 45.667 | 0.927 |
| | NNG | 13.167 | 0.287 | 0.667 | 0.039 | 6.000 | 0.207 | 15.500 | 0.345 | 7.833 | 0.198 | 6.167 | 0.149 | 14.667 | 0.336 | 25.167 | 0.605 | 2.667 | 0.067 | 4.500 | 0.161 | 23.667 | 0.512 |
| | NNU | 72.667 | 1.447 | 40.833 | 1.956 | 26.500 | 0.929 | 54.167 | 1.123 | 43.167 | 0.959 | 54.167 | 1.124 | 48.667 | 0.879 | 55.833 | 1.157 | 49.500 | 1.270 | 34.000 | 1.368 | 50.500 | 0.982 |

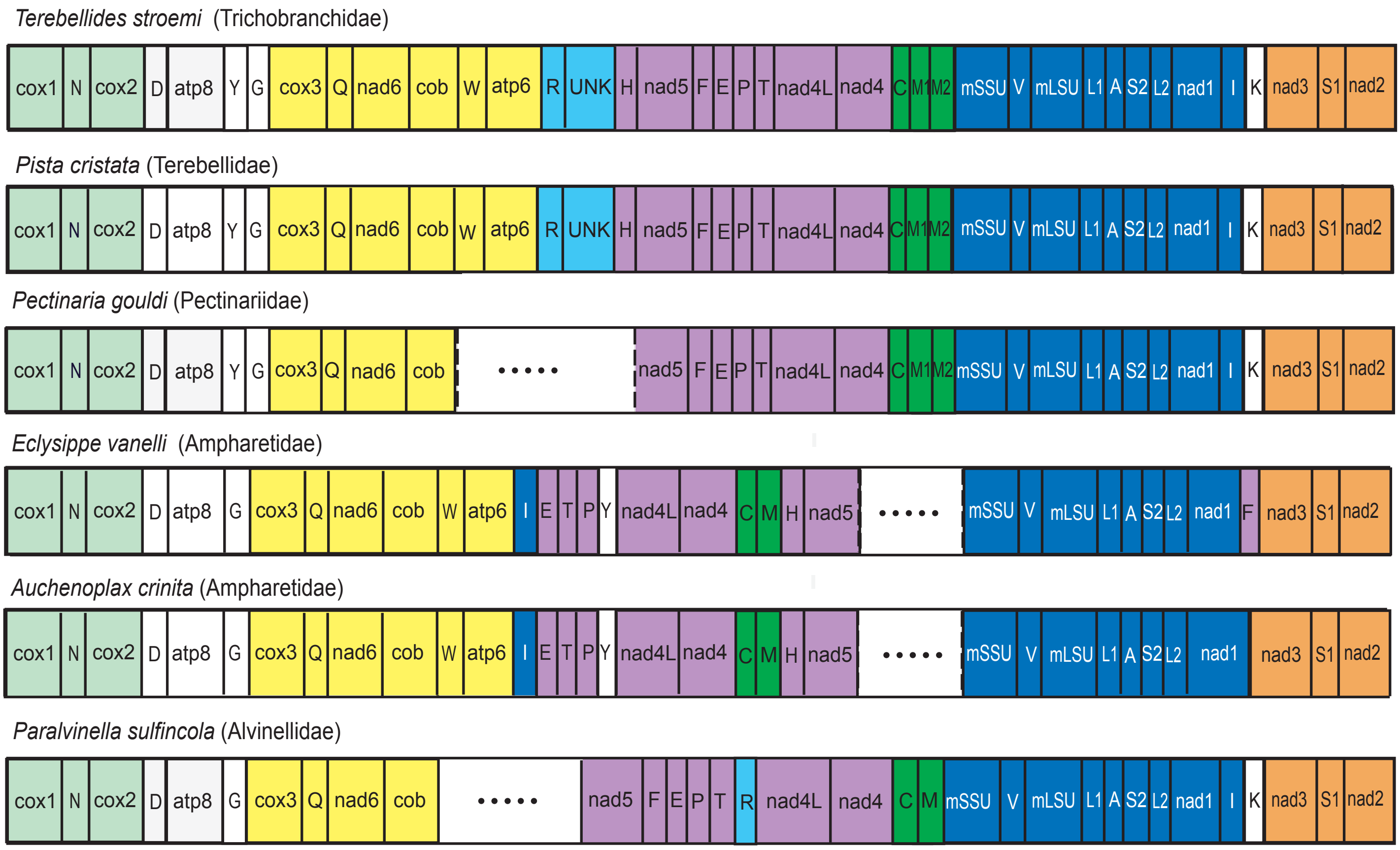


SUPPLEMENTRAY FIGURE 1. Mitochondrial gene order of six Terebelliformia worms. Different colors shows conserved gene clusters. Dots indicate missing regions.

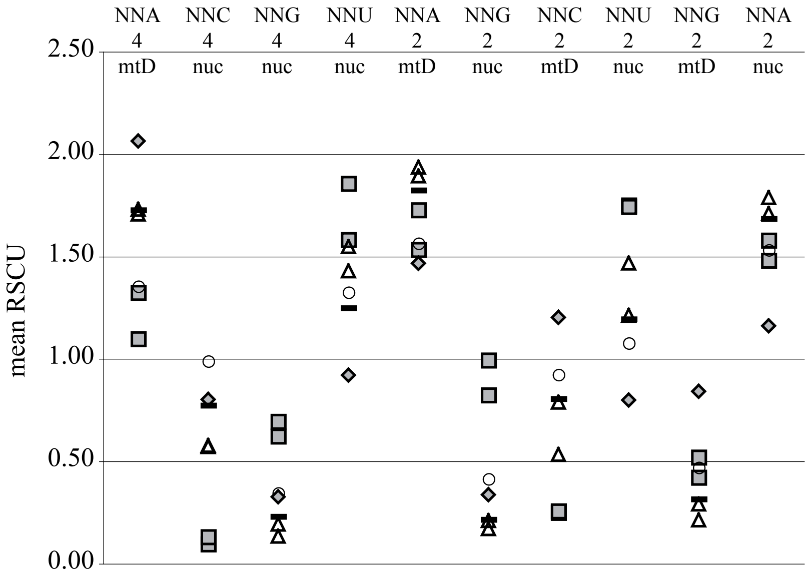


SUPPLEMENTRAY FIGURE 2. Analyses of of the relative synonymous codon usage in the mitochondrial protein-coding genes for Ampharetidae (grey squares), Alvinellidae (grey diamonds), Pectinariidae (open circles), Trichobranchidae and Terebellidae (both open triangles) as well as the mean values of outgroup taxa (black bar). NNA, NNC, NNG, and NNU indicate the 3rd position of the codon, 2 and 4 if the codon was 2-fold or 4-fold degenerated and mtD and nuc if the corresponding tRNA is located in the mitochondrial or nuclear genome, respectively.
