# Supplementary material for: Detecting the symplesiomorphy trap: a multigene phylogenetic analysis of terebelliform annelids
Source: BMC Evol Biol. 2011 Dec 20;11:369. doi: 10.1186/1471-2148-11-369 (PMC3282830; doi:10.1186/1471-2148-11-369)
Supplement: Additional file 2 — Analyses with increased taxon sets. This file provides a summary of datasets, analyses and results with more than 17 taxa. [file 1471-2148-11-369-S2.PDF]

Online Appendix 2. Summary of the datasets, analyses and results with more than 17 taxa.

Because taxon sampling was limited for mitochondrial genome sequences, we also conducted analyses of nuclear ribosomal gene data using a more comprehensive taxon sampling. Supplementary Table 5 lists taxa, gene sequences and GenBank accession numbers employed in this study for the rRNA datasets with more taxa. Sequences were aligned as described in the manuscript and subsequently corrected by hand in GeneDoc (Nicholas and Nicholas 1997). Ambiguous positions were determined using GBlocks (Castresana 2000) with default settings expect the allowed gap positions, which were set to "with half". The combined datasets comprised only taxa for which both 18S and 28S data were available with some composite OTUs (see Supplementary Table 5). Thus, we constructed three datasets, one comprising 61 taxa and consisting only of 18S rRNA data with 1,361 nucleotide positions as well as two others with 32 taxa and either only 28S rRNA data with 1,730 positions or 18S and 28S rRNA combined with 3,091 positions.

Similar BI and ML analyses as described in the manuscript were conducted on these three datasets. The parallel version of MrBayes 3.1 (Altekar et al. 2004, Huelsenbeck and Ronquist 2001, Ronquist and Huelsenbeck 2003) was used with specified models for the separate analyses (GTR+I+Γ for both genes) and the partitioned analysis of combined data. Each dataset ran for $2*10^6$ generations, with trees being sampled every 250 generations. The *burnin* was set to 1,200 trees for 28S and 1,600 for 18S and combined data. For the ML analyses the chosen models were TrN+I+Γ for 18S and GTR+I+Γ for 28S and the combined dataset. The reliability of phylogenetic nodes was estimated by 100 bootstrap replicates using the RAxML web-server (http://phylobench.vital-it.ch/raxml-bb/. Stamatakis et al. 2008).

Supplementary Figure 3 shows the 32-taxa trees reconstructed from the nuclear ribosomal genes (A - 28S; B -18S and 28S). Both trees reveal a topology of Terebelliformia consistent with the 17-taxa nuclear dataset except that Pectinariidae is sister to all other terebelliform taxa and, thus, monophyly of Terebelliformia is recovered. Among these five families, only the sistergroup relationship of Ampharetidae and Alvinellidae was well supported (BS: 93 for 28S/90 for combined; PP: 1.00 for both).

Further increasing the number of taxa by using only 18S rRNA data and excluding more of the positions in comparison to the combined analyses did not increase resolution (Supplementary Figure 4). For example, the analyses suggested paraphyly of Terebelliformia with the sipunculid *Phascolopsis gouldii* dropped into the Ampharetidae clade. Pectinariidae was close to Alvinellidae and Terebellidae was associated with Trichobranchidae, but both with weak nodal support (BS: 53; PP: 0.72 for Pectinaridae/Alvinellidae, lower than 50% for Terebellidae/Trichobranchidae). Furthermore, constraining Pectinariidae as being sister to all other Terebelliformia, as it was in most other analyses, placed Trichobranchidae as sister to Ampharetidae plus Alvinellidae. Also in previous analyses based only on 18S rRNA data the power to resolve deep annelid relationship was limited (e.g., Bleidorn et al. 2003, Halanych and Janosik 2006, Hall et al. 2004, McHugh 2005, Struck et al. 2002). Generally, it can be concluded that using 18S rRNA data alone is problematic to resolve phylogenetic relationships beyond the family level in Annelida.

References

Altekar G, Dwarkadas S, Huelsenbeck JP, Ronquist F. 2004. Parallel Metropolis-coupled Markov chain Monte Carlo for Bayesian phylogenetic inference. Bioinformatics, 20:407-415.

Bleidorn C, Vogt L, Bartolomaeus T. 2003. New insights into polychaete phylogeny (Annelida) inferred from 18S rDNA sequences. Mol. Phylogenet. Evol., 29:279-288.

Castresana J. 2000. Selection of conserved blocks from multiple alignments for their use in phylogenetic analysis. Mol. Biol. Evol., 17:540-552.

Halanych KM, Janosik AM. 2006. A review of molecular markers used for Annelid phylogenetics. Integr. Comp. Biol., 46:533-543.

Hall KA, Hutchings PA, Colgan DJ. 2004. Further phylogenetic studies of the Polychaeta using 18S rDNA sequence data. J. Mar. Biol. Ass. UK, 84:949-960.

Huelsenbeck JP, Ronquist F. 2001. MRBAYES: Bayesian inference of phylogenetic trees. Bioinformatics, 17:754-755.

McHugh D. 2005. Molecular systematics of polychaetes (Annelida). Hydrobiologia, 535/536:309 - 318.

Nicholas KB, Nicholas HBj. 1997. GeneDoc: a tool for editing and annotating multiple sequence alignments., Distributed by the author.

Ronquist F, Huelsenbeck JP. 2003. MRBAYES 3: Bayesian phylogenetic inference under mixed models. Bioinformatics, 19:1572-1574.

Stamatakis A, Hoover P, Rougemont J. 2008. A rapid bootstrap algorithm for the RAxML web-servers. Syst. Biol., 75:758-771.

Struck TH, Hessling R, Purschke G. 2002. The phylogenetic position of the Aeolosomatidae and Parergodrilidae, two enigmatic oligochaete-like taxa of the 'Polychaeta', based on molecular data from 18SrDNA sequences. J. Zool. Syst. Evol. Res., 40:155-163.

SUPPLEMENTARY TABLE 5. List of taxa and genes used for the phylogenetic analyses based on more than 17 taxa and using only 18S and 28S data. Accession numbers of determined sequences in bold. Voucher number and locality are provided with accession number.

Taxon	18S rDNA	28S rDNA	Locality
**Terebelliformia**			
Alvinellidae			
Alvinella pompejana Desbruyères & Laubier, 1980	AM159573		
Alvinella caudata Desbruyères & Laubier, 1986	AM159574		
Paralvinella grasslei Desbruyères & Laubier, 1982	AY577886		
	AM159575		
Paralvinella palmiformes Desbruy. & Laubier, 1986	AF168747		
		**JN936479**	47°56.947'N/129°05.878'W Juan de Fuca (Canada)
Paralvinella pandorae Desbruy. & Laubier, 1986	AM159576		
	**JN936460**		47°56.945'N/129°05.881'W Juan de Fuca (Canada)
Paralvinella sulfincola Desbruy. & Laubier, 1993	**JN936461**		47°57.001'N/129°05.851'W Juan de Fuca (Canada)
Ampharetidae			
Amphisamytha bioculata (Moore, 1906)	**JN936466**	**JN936486**	36°51.433'N/121°51.438'W Monterey Bay, CA (USA)
Amphisamytha galapagensis Zottoli, 1983	AM159578		
Anobothrus gracilis (Malmgren, 1866)	**JN936468**	**JN936487**	41°09.869'N/70°25.04'W
Anobothrus gracilis (Malmgren, 1866)	AY611458		
Auchenoplax crinita Ehlers, 1887	DQ790077	DQ790026	
Eclysippe vanelli (Fauvel, 1936)	**JN936467**	**JN936489**	63°30.84'N/10°25.01'E Storgrunnen (Norway)
Glyphanostomum sp.	DQ209225		
Paralysippe annectens (Moore, 1923)	**JN936464**	**JN936488**	36°55.182'N/121°54.279'W
Samytha sexcirrata (M. Sars, 1856)	**JN936465**	**JN936485**	39°59.949'N/70°41.618'W
Melinna sp.	AY611459		
Pectinariidae			
Pectinaria dodeka Hutchings & Peart, 2002	AB106263		
Pectinaria gouldi (Verrill, 1873)	DQ790091	DQ790054	
Pectinaria granulata (Linnaeus, 1767)	AY577890		
Pectinaria koreni (Malmgren, 1866)	**JN936463**		54°10´N/7°50´E Helgoland (Germany)
Pectinaria regalis Verrill, 1901	AY040698		
Terebellidae			
Amphitrite ornata (Leidy, 1855)	DQ790074	DQ790022	
Amphitritides harpa Hutchings & Glasby, 1988	AB106260		
Amphitritides gracilis (Grube, 1860)	AF508115		
Artacama proboscidea Malmgren, 1866	AY344666		
Eupolymnia nesidensis (Delle Chiaje, 1828)	AY611460		
Lanice conchilega (Pallas, 1766)	**JN936470**	**JN936484**	55°01.34'N/8°26.13'E List, Sylt (Germany)
Lanice conchilega (Pallas, 1766)	X79873.1		
Loimia medusa (Savingy, 1818)	AY040690		
Lysilla sp.	**JN936474**	**JN936482**	36°49.847'N/122°01.729°W
Lysilla pacifica Hessle, 1917	AB106259		
Nicolea zostericola (Oersted, 1844)	**JN936469**		54°10.37'N/7°53.46'E

			Helgoland (Germany)
Pista cristata (Mueller, 1776)	AY611461	DQ790057	
Pista sp.	AB106261		
Polycirrus cf. *eximius* (Leidy, 1855)	**JN936475**	**JN936481**	40°35.596'N/71°31.911'W
Streblosoma sp.	**JN936471**	**JN936483**	39°59.949'N/70°41.618'W
Terebella lapidaria Linnaeus, 1767	**JN936476**		Seawater aquarium Free University of Berlin
Thelepinae sp.	**JN936472**		37°45.936'N/123°11.615'W Farallon, CA (USA)
Thelepus crispus Johnson, 1901	**JN936473**	**JN936480**	48°27.178'N/122°57.760'W Cattle Point, WA (USA)
Thelepus cincinnatus (Fabricius, 1780)	AY611462		
Trichobranchidae			
Terebellides californica Williams, 1984	**JN936462**	**JN936478**	36°55.182'N/121°54.279'W
Terebellides stroemi M. Sars, 1835	DQ790094	DQ790066	
Terebellides stroemi M. Sars, 1835	AY611463		
Terebellides stroemi M. Sars, 1835	AY577893		
**Outgroup**			
Sipuncula			
Phascolopsis gouldi (Pourtalès, 1851)	AF342796	AF342795	
Echiura			
Urechis caupo Fisher & MacGinitie, 1928	AF119076	AF519268	
Clitellata			
Lumbricus terrestris Linnaeus, 1758	AJ272183	DQ790041	
Hirudo medicinalis Linnaeus, 1758	Z83752	AY364866	
Maldanidae			
Clymenella torquata (Leidy, 1855)		DQ790030	
Clymenura clypeata (Saint-Joseph, 1894)	AF448152		
Orbiniidae			
Orbinia swani Pettibone, 1957	DQ790087	DQ790048	
Leitoscoloplos fragilis (Verrill, 1873)	AY532360	EU418863	
Nereididae			
Nereis vexillosa Grube, 1851	DQ790083	DQ790043	
Nephtyidae			
Nephtys longosetosa (Oersted, 1842)	DQ790082	DQ790042	
Spionidae			
Prionospio dubia Maciolek, 1985	EU418859	EU418867	
Chaetopteridae			
Chaetopterus variopedatus (Renier, 1804)	U67324	AY145399	
Sabellaridae			
Sabellaria cementarium Moore, 1906	AY732223	AY732226	
Sabellidae			
Schizobranchia insignis Bush, 1905	AY732222	AY732225	
Siboglinidae			
Riftia pachyptila Jones, 1981	AF168739	Z21534	
Cirratulidae			
Tharyx sp.	**JN936459**	**JN936477**	41°09.869'N/70°25.040'W

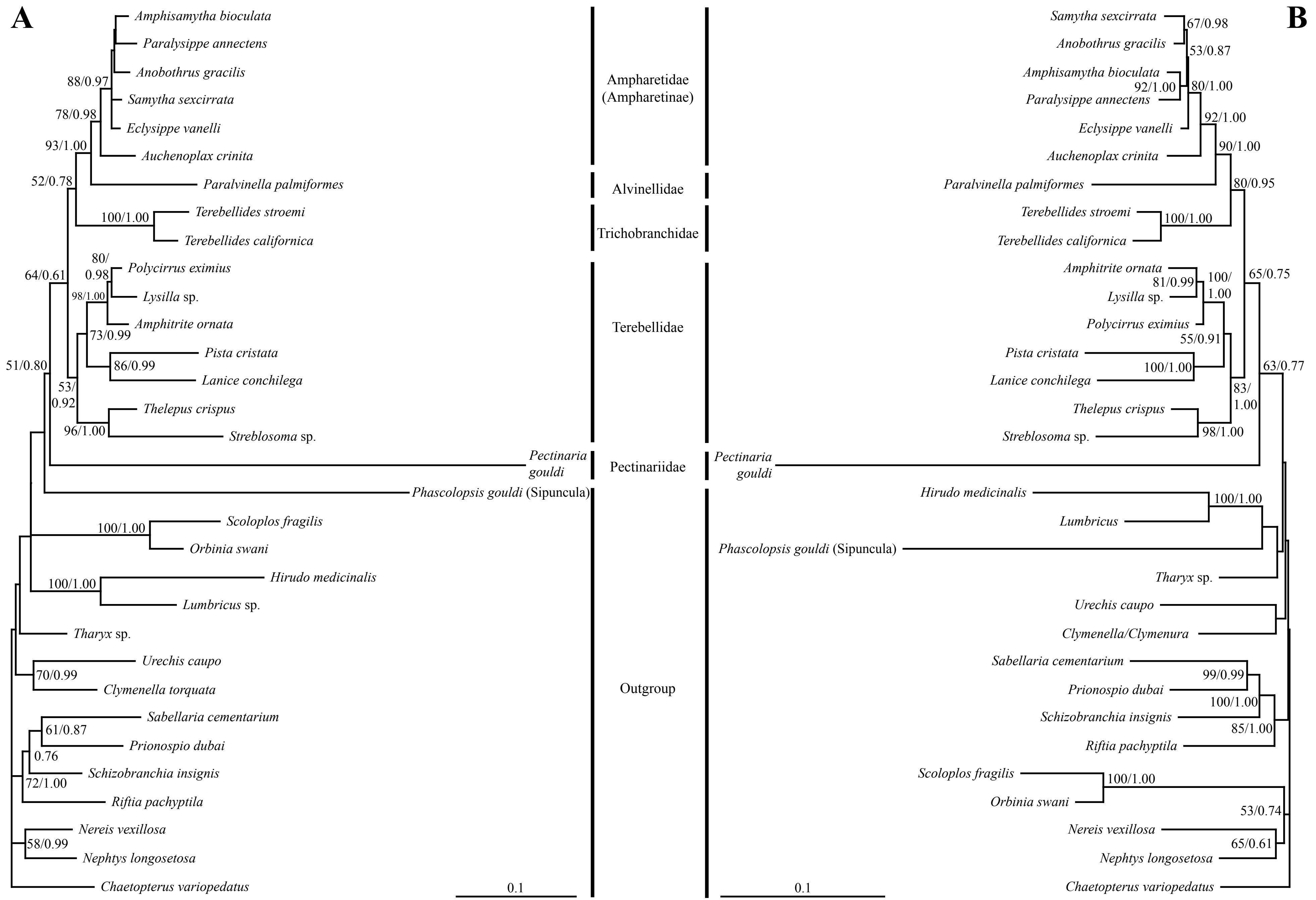

SUPPLEMENTARY FIGURE 3. Phylogenetic reconstructions using 28S and 18S/28S combined datasets with 32 taxa. (A) 28S ML tree. (B) Combined ML tree. All trees represent identical topologies of both ML and partitioned BI. Nodal support values are given at branches with ML bootstrap values first and PP of the partitioned BI second. < 50% are not shown.

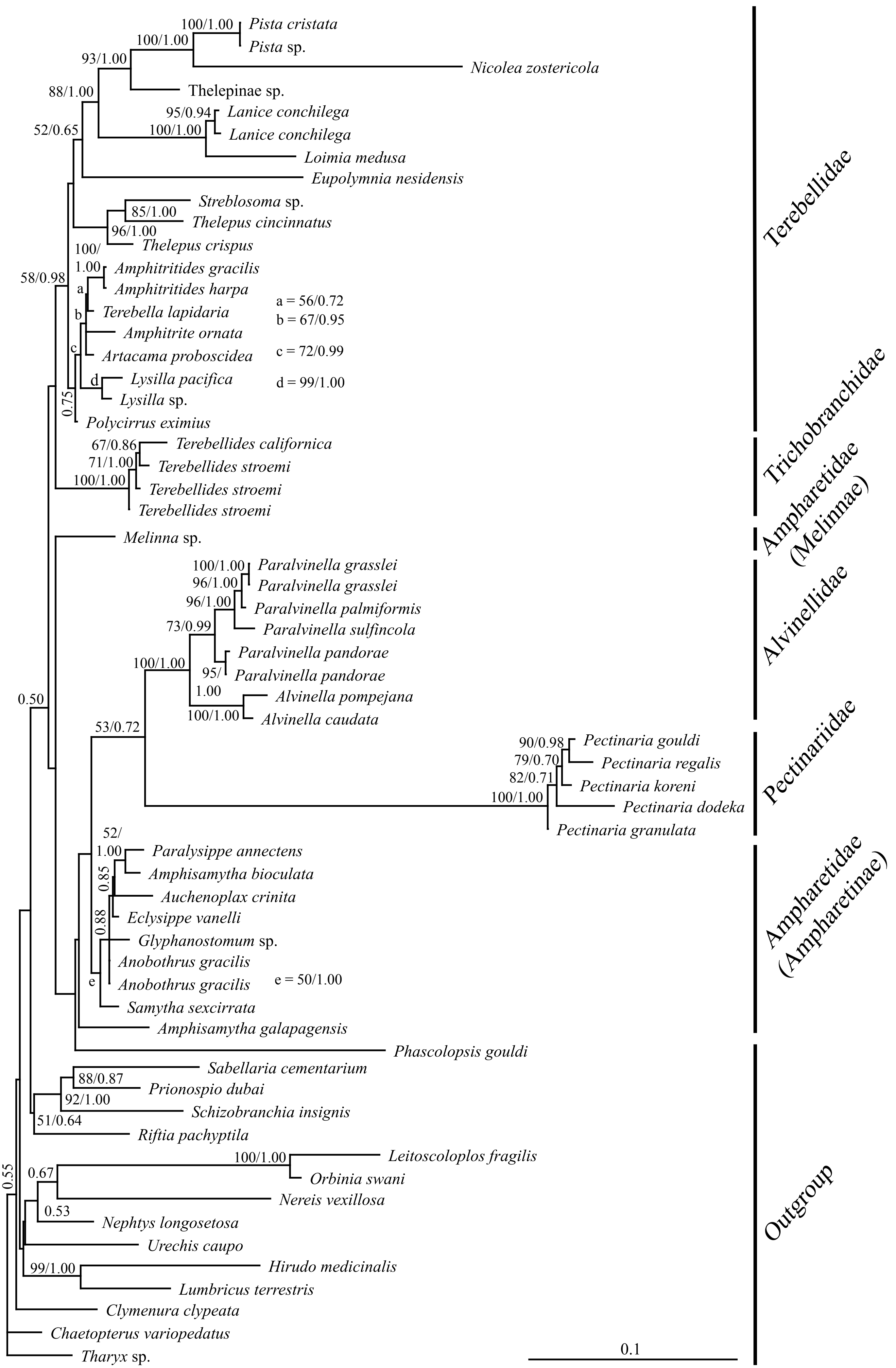

SUPPLEMENTARY FIGURE 4: The best ML tree, also identical with the BI, based on the 18S datasets with 61 taxa. Bootstrap values and PP are shown at the nodes in order. A dash indicates < 50%.
