# Supplementary material for: Detecting the symplesiomorphy trap: a multigene phylogenetic analysis of terebelliform annelids
Source: BMC Evol Biol. 2011 Dec 20;11:369. doi: 10.1186/1471-2148-11-369 (PMC3282830; doi:10.1186/1471-2148-11-369)
Supplement: Additional file 3 — Best ML trees of the amino acid datasets with 17 taxa. This file provides a supplementary figure showing the best tree of ML and BI analyses based on mitochondrial and combined amino acid datasets. [file 1471-2148-11-369-S3.PDF]

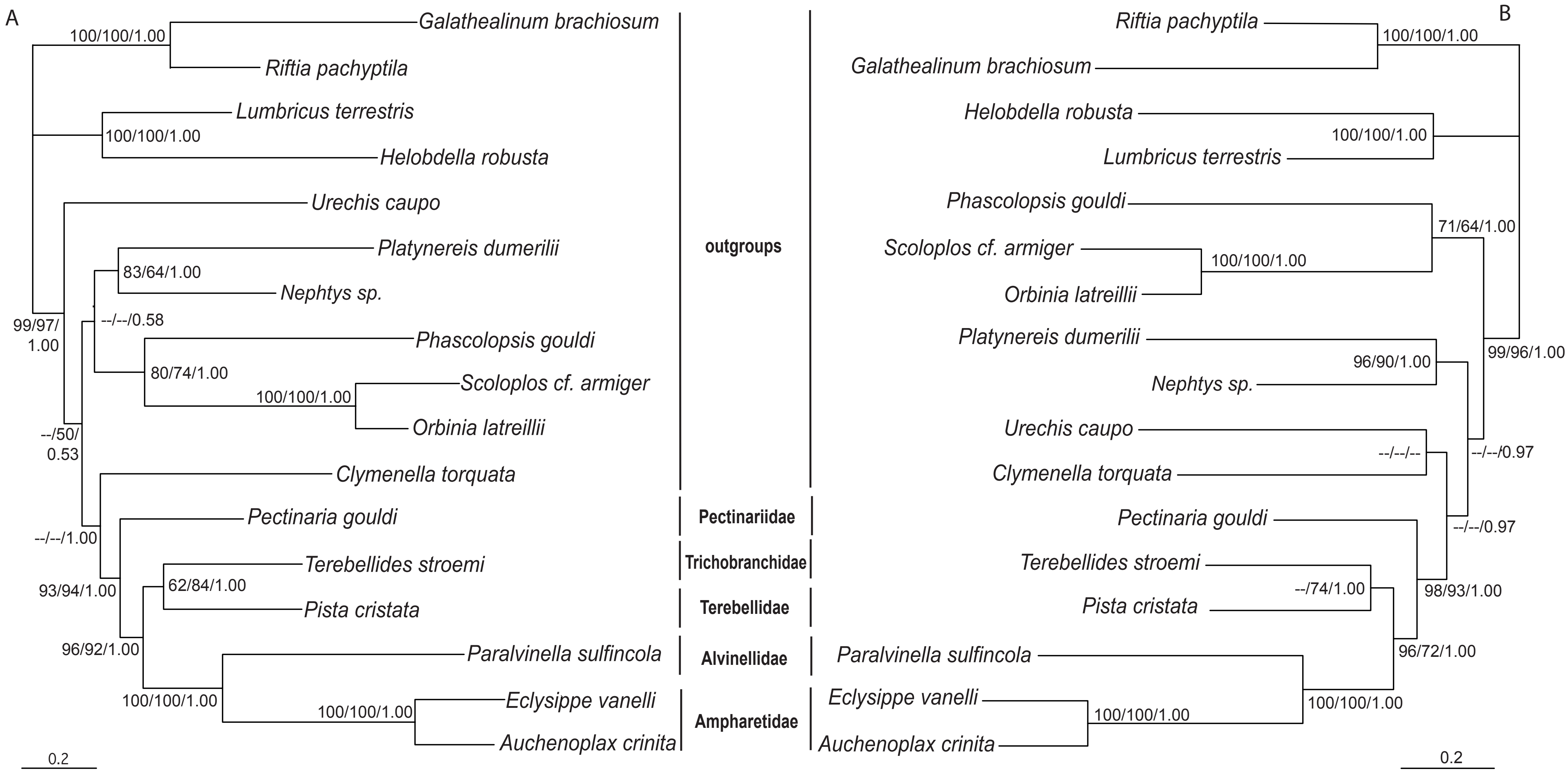

Online Appendix 3: The best non-partitioned ML, also identical with the partitioned ML and BI trees regarding terebelliform relationships, based on mitochondrial (A) and combined amino acid datasets (B). Bootstrap values and PP are shown at the nodes in the order non-partitioned ML, partitioned ML and BI. A dash indicates < 50%.
