# Supplementary material for: Detecting the symplesiomorphy trap: a multigene phylogenetic analysis of terebelliform annelids
Source: BMC Evol Biol. 2011 Dec 20;11:369. doi: 10.1186/1471-2148-11-369 (PMC3282830; doi:10.1186/1471-2148-11-369)
Supplement: Additional file 4 — Compositional heterogeneity of the 3rd positions. This file provides a supplementary figure showing the analyses of compositional heterogeneity of 3rd positions included in the mitochondrial dataset as well as of only the 3rd positions of the mitochondrial protein-coding genes. [file 1471-2148-11-369-S4.PDF]

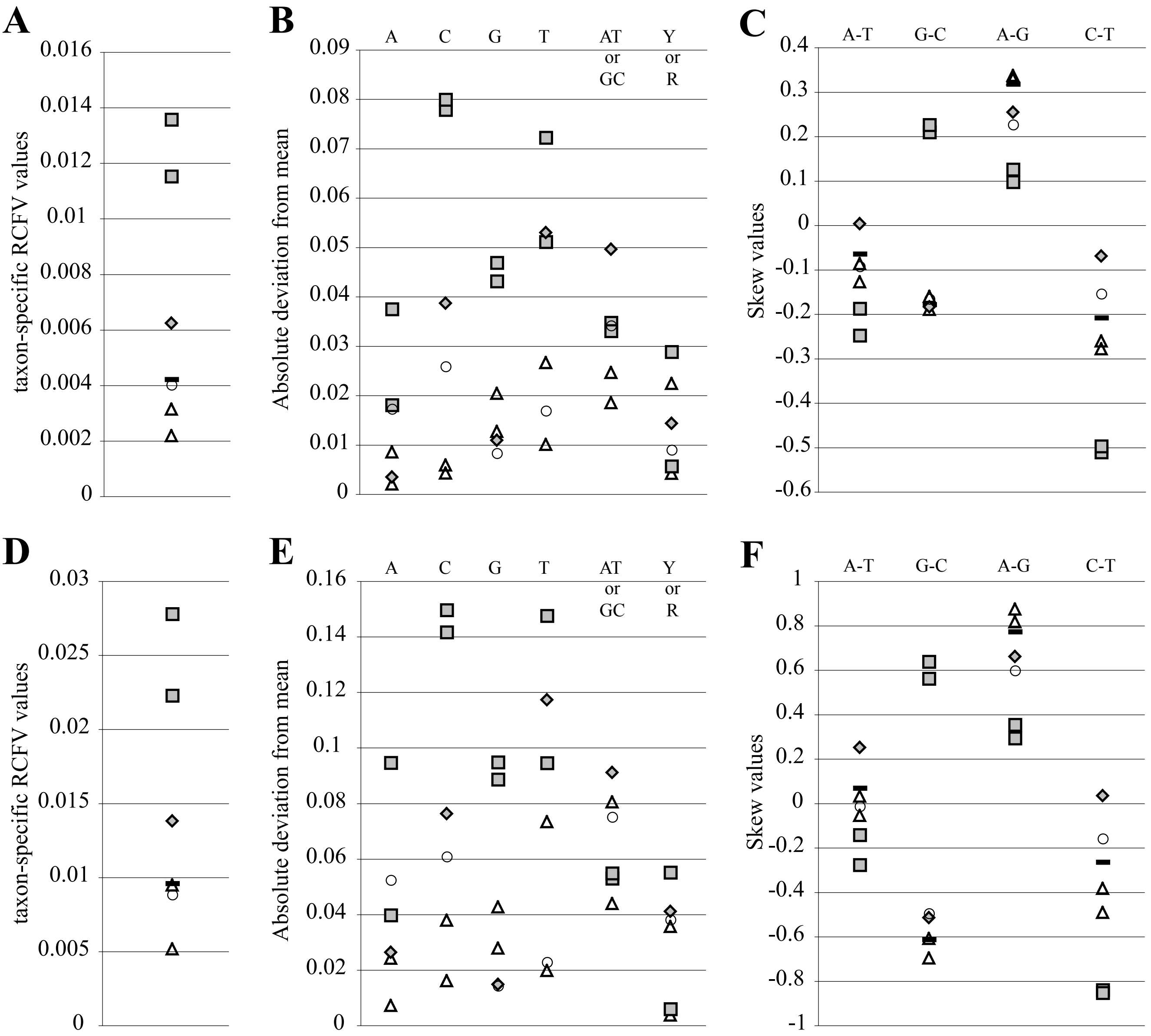


**Online Appendix 4:** The analyses of compositional heterogeneity of 3rd positions included in the mitochondrial dataset (A-C) as well as of only the 3rd positions of the mitochondrial protein-coding genes (D-F) for Ampharetidae (grey squares), Alvinellidae (grey diamonds), Pectinariidae (open circles), Trichobranchidae and Terebellidae (both open triangles) as well as the mean values of the outgroup taxa (black bar). (A, D) Taxon-specific relative composition frequency variability (RCFV). (B, E) Absolute deviation from mean frequency for adenine (A), cytosine (C), guanine (G), and thymine (T) as well as combinations of adenine/thymine (AT) or guanine/cytosine (GC) and of pyrimidines (Y) or purines (R). (C, F) Skew values within the combinations adenine/thymine (A-T), guanine/cytosine (G-C), purines (A-G) and pyrimidines (C-T).
