# Supplementary material for: Detecting the symplesiomorphy trap: a multigene phylogenetic analysis of terebelliform annelids
Source: BMC Evol Biol. 2011 Dec 20;11:369. doi: 10.1186/1471-2148-11-369 (PMC3282830; doi:10.1186/1471-2148-11-369)
Supplement: Additional file 5 — Analyses using time-heterogeneous models. This file provides a supplementary figure showing the results of the PHASE analyses using 3, 6 or 9 compositional vectors, respectively, for both the mitochondrial and nuclear dataset. [file 1471-2148-11-369-S5.PDF]

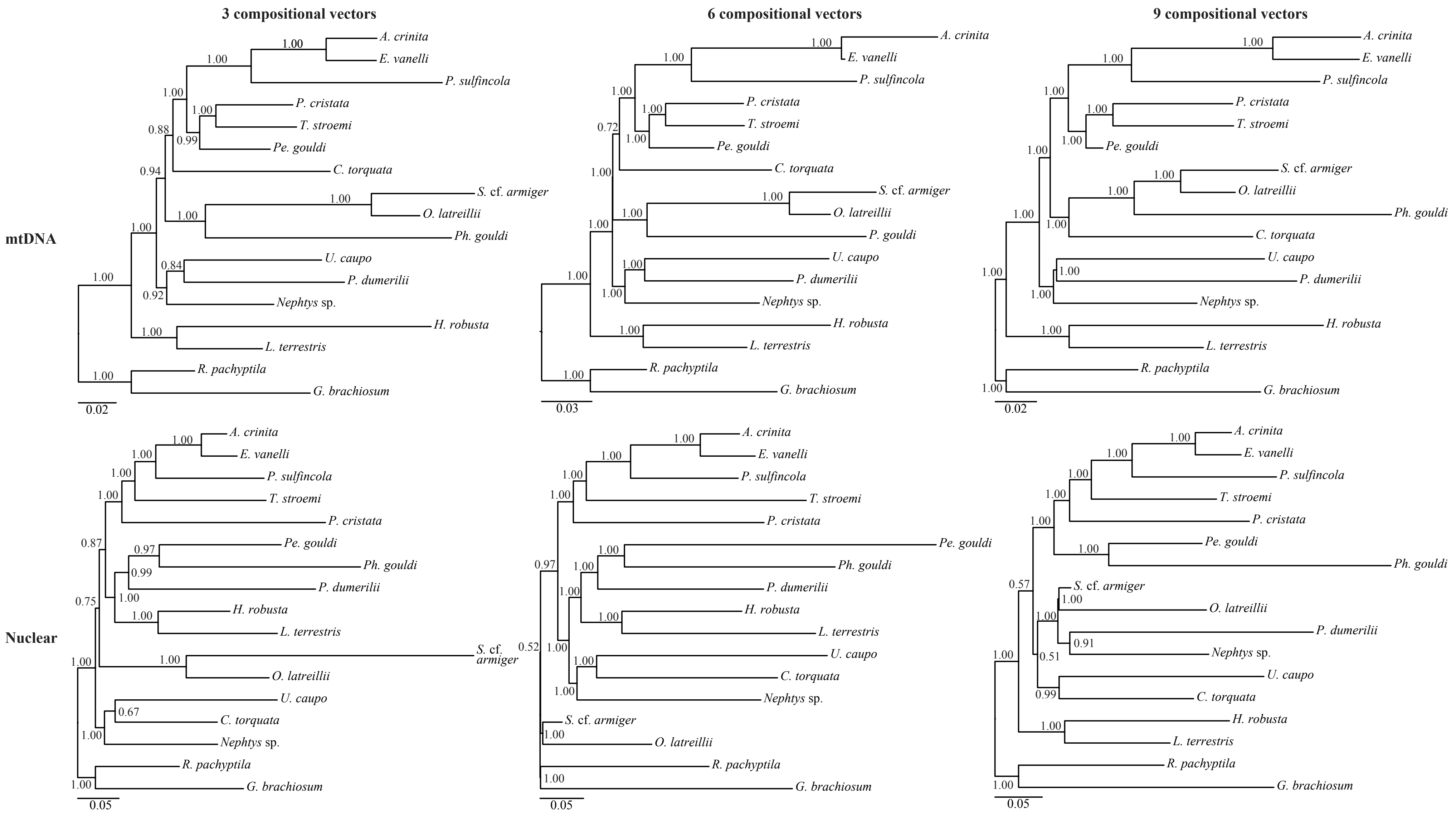


**Online appendix 5:** Phylogenetic reconstructions using PHASE with 3, 6 or 9 compositional vectors, respectively, for both the mitochondrial and nuclear nucleotide datasets with 17 taxa. The majority rule consensus trees are shown and posterior probabilities are given at the branches.
